# Effects of non-invasive brain stimulation on balance control in patients with multiple sclerosis: a systematic review and meta-analysis

**DOI:** 10.3389/fneur.2025.1696343

**Published:** 2025-10-10

**Authors:** Longtao Zhang, Yanxiao Sun, Zhihao Diao, Yifan Jiang, Yufei Hu, Yuxia Ma

**Affiliations:** ^1^Acupuncture and Tuina College of Shandong University of Traditional Chinese Medicine, Jinan, Shandong, China; ^2^The First Clinical Medical College of Shandong University of Traditional Chinese Medicine, Jinan, Shandong, China; ^3^Key Laboratory of Traditional Chinese Medicine Classical Theory, Ministry of Education, Shandong University of Traditional Chinese Medicine, Jinan, Shandong, China

**Keywords:** non-invasive brain stimulation, multiple sclerosis, balance control, meta-analysis, randomized controlled trials, systematic review

## Abstract

**Objective:**

This study aims to evaluate the effects of non-invasive brain stimulation (NIBS) on balance control in patients with multiple sclerosis (MS) through a systematic review and meta-analysis. The goal is to identify the most effective treatment strategies and provide valuable evidence for clinical decision-making.

**Methods:**

PubMed, Web of Science, Embase, Scopus, Cochrane Library, ClinicalTrials.gov and CNKI Scholar were searched for randomized controlled trials (RCTs) on the effects of NIBS on balance control in patients with MS. The search period was from the inception of each database to August 11, 2025. The Timed Up and Go test (TUG) and the Berg Balance Scale (BBS) are the primary outcome measures, with adverse events being the secondary outcome measure. Two researchers independently performed literature screening, data extraction and quality assessment. The quality of the included trials was assessed using the Cochrane Risk of Bias tool (RoB 2.0) and the GRADE proGDT software was used to evaluate the evidence grading recommendation standards (GRADE) for outcomes. Data were analyzed using RevMan 5.4 and StataMP 18 software. For binary variables, the effect size is measured using the risk ratio (RR), while for continuous variables, the effect size is measured using the mean difference (MD), with a 95% confidence interval (CI). Heterogeneity was explored through subgroup analysis, meta-regression, and sensitivity analysis to assess the robustness of the results. A funnel plot was constructed, and Egger’s test was performed to evaluate potential publication bias.

**Results:**

A total of 17 RCTs with 514 patients were included. Meta-analysis showed that NIBS can shorten the time taken for TUG [MD = −1.03, 95% CI (−1.86, −0.20)] and improve BBS scores [MD = 3.35, 95% CI (1.31, 5.39)], indicating that NIBS may improve both dynamic and static balance. Subgroup analysis revealed that interventions lasting ≥4 weeks were associated with a reduction in TUG completion time and an increase in BBS scores. Furthermore, transcranial direct current stimulation (tDCS) demonstrated favorable effects on both TUG and BBS outcomes, while evidence supporting the efficacy of repetitive transcranial magnetic stimulation (rTMS) remained limited. Although adverse events such as itching, warmth, tension, and fatigue were reported in NIBS group, these were generally mild and transient.

**Conclusion:**

This study suggests that NIBS may serve as an effective adjunctive therapy for balance rehabilitation in patients with MS, showing benefits in both dynamic and static balance. However, its application is accompanied by mild and transient adverse effects, necessitating a careful balance between efficacy and safety in clinical practice. The current evidence is limited by heterogeneity among included studies and short follow-up durations. Future research should focus on large-scale, high-quality RCTs to further validate the long-term efficacy of NIBS, optimize stimulation parameters, and promote the development of individualized treatment strategies.

**Systematic review registration:**

https://www.crd.york.ac.uk/prospero/, identifier: CRD420251121717.

## 1 Introduction

Multiple sclerosis (MS) is a chronic, immune-mediated disease characterized by inflammatory demyelination in the central nervous system, and is one of the leading causes of disability in young adults worldwide (1, 2). The clinical symptoms include limb movement disorders, visual disturbances, sensory abnormalities, and cognitive dysfunction, with a high rate of disability and progressive patients may face paralysis, blindness, cognitive decline and other consequences (1). Ninety percent of MS patients will experience significant motor dysfunction within 25 years of diagnosis, leading to balance disorders, gait abnormalities, and reduced quality of life (3). According to the latest MS global monitoring data, approximately 2.8 million people were affected by MS in 2020, and the prevalence is increasing worldwide. The pathological changes in MS include demyelination, axonal loss, and neurodegeneration, which particularly affect the neural networks involved in balance control, leading to impairments in sensory integration and motor output (4, 5). Balance dysfunction in MS significantly increases the risk of falls, limits mobility, and reduces quality of life. Therefore, restoring or improving balance is crucial for the management of patients with MS (6, 7).

Currently, the treatment of MS primarily relies on disease-modifying therapies (DMTs) and immunomodulatory, but the efficacy of drugs is limited and associated with certain adverse effects (8). Traditional rehabilitation methods such as physical therapy and exercise programs have shown benefits in improving balance and mobility in MS patients (9, 10). However, the effectiveness of these interventions is often constrained by variability in disease severity, patient adherence, and response (11).

In recent years, non-invasive brain stimulation (NIBS), such as transcranial direct current stimulation (tDCS) and repetitive transcranial magnetic stimulation (rTMS), has emerged as a promising adjunct to conventional rehabilitation strategies due to its non-invasiveness, safety, and good tolerability (12). Studies have shown that the core mechanism of tDCS involves modulating the functional balance of distributed neural networks and enhancing neuroplasticity (13). Specifically, anodal tDCS has been found to improve motor performance during balance tasks and enhance overall balance ability (14–16). NIBS may improve motor learning and functional recovery by modulating cortical excitability and promoting neuroplasticity, particularly by enhancing balance control through its effects on sensorimotor networks (17–19).

Previous studies (20, 21) have mainly focused on spasticity, walking speed, or overall motor function improvement, with relatively few systematic and quantitative analyses focused specifically on balance control as the primary outcome. Due to high heterogeneity among studies, insufficient stratification of stimulation targets, and methodological differences, the exact efficacy of NIBS on balance function in MS patients has not been clearly established. In particular, there is still a lack of consensus regarding the modulatory effects of various stimulation parameters, such as stimulation site, duration, and intensity. Therefore, this study aims to overcome these limitations through a more comprehensive literature search and systematic quantitative analysis. We will not only comprehensively evaluate the overall effect of NIBS on balance function in MS but also explore the potential sources of the aforementioned heterogeneity in depth through detailed subgroup analyses (stratified by NIBS type, MS subtype, stimulation parameters, intervention duration, etc.) and meta-regression, clarifying the relationship between different stimulation parameters and efficacy, in order to provide more precise and valuable evidence-based support for clinical decision-making and future research design in this field.

## 2 Materials and methods

### 2.1 Study design and registration

This systematic review and meta-analysis have been registered with PROSPERO (CRD420251121717). This study followed the Preferred Reporting Project for Systematic Reviews and Meta-Analyses (PRISMA) 2020 guidelines (22, 23) (Supplementary Table S1).

### 2.2 Search strategy

Databases searched included PubMed, Web of Science, Embase, Scopus, Cochrane Library, ClinicalTrials.gov and CNKI Scholar. The search period was from the inception of each database to August 11, 2025. The search utilized both controlled vocabulary and free-text terms. The following keywords were used for the search: “multiple sclerosis,” “non-invasive brain stimulation,” “transcranial magnetic stimulation,” “transcranial direct current stimulation,” “transcranial alternating current stimulation,” “theta burst transcranial magnetic stimulation,” “balance,” and “randomized controlled trials,” among others. See Supplementary Table S2 for detailed search strategies.

### 2.3 Inclusion and exclusion criteria

#### 2.3.1 Inclusion criteria

1. Population: Adults diagnosed with MS according to McDonald criteria (24), regardless of sex or disease duration.
2. Intervention: Received any of the following interventions, alone or in combination: rTMS, tDCS, theta burst stimulation (TBS), combined rehabilitation training (such as balance training, aerobic exercise, virtual reality training), or other NIBS techniques.
3. Comparison: Sham stimulation, conventional rehabilitation, or no treatment.
4. Outcomes: The primary outcome was at least one measure of balance function, specifically the Timed Up and Go (TUG) test or the Berg Balance Scale (BBS). The secondary outcome was adverse events (AEs).
5. Study design: Published randomized controlled trials (RCTs) literature.

#### 2.3.2 Exclusion criteria

1. Studies with unavailable full text or incomplete reporting.
2. Studies using invasive brain stimulation techniques.
3. Studies with concurrent treatments that could affect balance (unless the same treatment was applied to the control group).
4. Non-RCT studies or conference abstracts.

### 2.4 Literature screening and data extraction

Two researchers independently managed the literature using EndNote 20 software. After deduplication, the title, abstract and full text were read, and the literature that met the inclusion criteria was screened and cross-checked. Disagreements were resolved through discussion with a third researcher. The data of the literature were extracted using a pre-formulated Excel table, and the extracted information included: title, first author, year of publication, sample size, age, course of disease, Expanded Disability Status Scale (EDSS), MS subtype, intervention measures, intervention protocol, stimulation site, stimulation intensity, duration of treatment, outcomes and AEs.

### 2.5 Quality assessment of included studies

Two researchers independently assessed the quality of the studies using the Cochrane Risk of Bias tool (RoB 2.0) (25) in the following domains: randomization process, deviations from intended interventions, missing outcome data, measurement of the outcome, and selection of the reported result. The results were categorized as “low risk,” “some concerns,” or “high risk.” Disagreements were resolved through discussion with a third researcher.

### 2.6 Certainty of evidence

Two researchers assessed the certainty of evidence for the outcomes using the GRADE approach with GRADE pro GDT software (Grading of Recommendations, Assessment, Development and Evaluation) (26). The GRADE system evaluates the quality of evidence based on six domains: study design, risk of bias, inconsistency, indirectness, imprecision, and publication bias. The overall quality of evidence is classified into four levels: high, moderate, low, and very low.

### 2.7 Statistical analysis

Statistical analysis was performed using RevMan 5.4 and StataMP 18 software. For dichotomous variables (adverse events), the effect size was expressed as risk ratio (RR). For continuous variables, the mean difference (MD) was used to estimate the effect size. Both were reported with 95% confidence intervals (CI). If the 95% CI did not include zero, the difference between groups was considered statistically significant. Heterogeneity between studies was assessed using the *I*^2^ statistic. If *I*^2^ ≤ 50%, low heterogeneity was assumed, and a fixed-effects model was used for analysis. If *I*^2^ > 50%, high heterogeneity was indicated, and a random-effects model was employed. For the meta-analysis, the between-study variance was estimated using the DerSimonian–Laird method. When significant heterogeneity was detected, subgroup analysis or meta-regression was conducted to explore its potential sources. Meta-regression was performed for continuous moderators (e.g., mean age, baseline EDSS) using restricted maximum likelihood (REML) estimation in StataMP 18. Sensitivity analysis was carried out by excluding individual studies one by one to test the robustness of the combined results. A funnel plot combined with Egger’s test was used to assess potential publication bias. The significance level was set at *α* = 0.05.

## 3 Results

### 3.1 Literature search results

**Figure 1:**
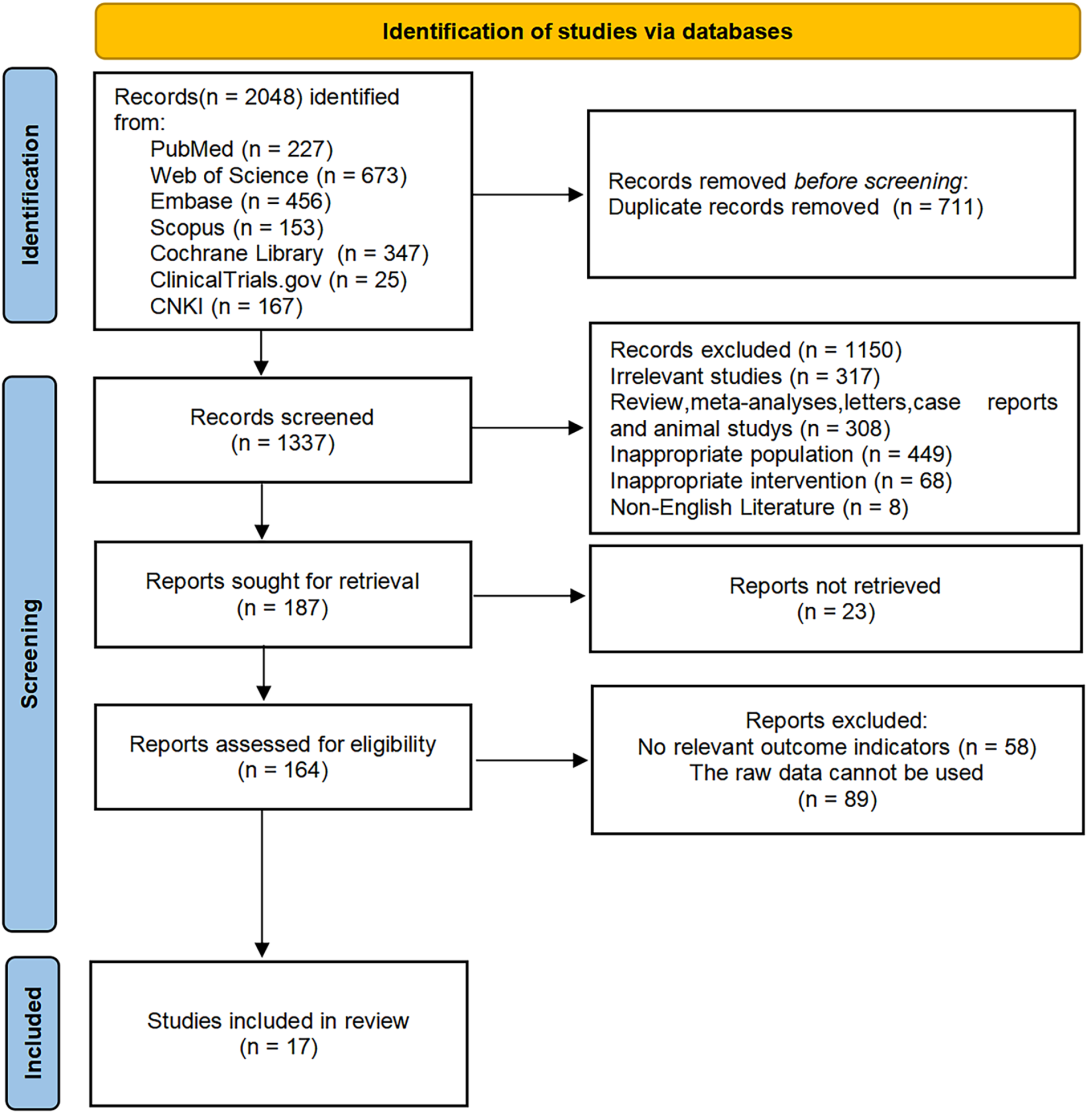
Flow chart of the study selection process.

A total of 2,048 relevant articles were identified through database searches. After removing duplicates, 1,337 articles remained. Based on the inclusion and exclusion criteria, 187 articles were selected for full-text review. After screening the full texts, 170 articles were excluded. Ultimately, 17 studies met the eligibility criteria and were included in the final analysis (Figure 1).

### 3.2 Basic characteristics of the included studies

A total of 17 RCTs (27–43) involving 514 MS patients were included in this study. Among these, 269 patients were assigned to the treatment group, and 245 patients were in the control group. The sample sizes of individual studies ranged from 16 to 64 participants, with a mean age range of 35 to 55 years. The duration of the interventions varied from 1 week to 6 weeks. Thirteen studies used tDCS, two studies used rTMS, one study used repetitive transspinal magnetic stimulation (rTSMS), and one study used intermittent theta burst stimulation (iTBS). The interventions in the control group were rehabilitation training or sham stimulation. The detailed characteristics of the included studies are presented in Tables 1, 2.

**Table 1:** Basic characteristics of the included studies.

Study	Country	Study type	Overall age	EDSS (T/C)	MS type	Treatment group					Control group					Outcome
						Sample size (n)	Age (years)	Gender (M/F)	Disease duration (year)	Intervention	Sample size (n)	Age (years)	Gender (M/F)	Disease duration (year)	Comparison	
Tramontano et al. (27)	Italy	RCT	51.75 ± 7.9	5.75 ± 0.89	SP PR	8	51.9 ± 3.83	2/6	18.13 ± 8.24	iTBS + vestibular rehabilitation (VR)	8	51.6 ± 10.91	3/5	18.75 ± 9.62	Sham iTBS + vestibular rehabilitation (VR)	BBS
Mohamed et al. (28)	Egypt	RCT	33.75 ± 6.7	3.385 ± 0.64	RR SP	15	31.6 ± 5.6	6/9	5.8 ± 1.7	rTMS + physiotherapy	15	35.9 ± 7.2	6/9	5.3 ± 1.3	rTMS + physiotherapy	BBS, AEs
Charehjou et al. (29)	Iran	RCT	39.35 ± 11.2	6.25 ± 0.18	NR	10	39.9 ± 12.38	3/7	NR	tDCS + virtual reality (VR) training	10	40.0 ± 10.57	3/7	NR	Virtual reality (VR) training	BBS
Baroni et al. (30)	Italy	RCT	53.69 ± 13.01	4.59 ± 0.61	RR PP SP	8	55.25 ± 15.15	4/4	11.13 ± 6.99	tDCS + TOCT	8	52.13 ± 11.31	4/4	11.13 ± 9.95	sham tDCS + TOCT	TUG, AEs
Mohammadkhanbeig et al. (31)	Iran	RCT	37.57 ± 7.61	3.6 ± 1.42	RR	9	37.44 ± 7.89	0/9	9.44 ± 4.30	tDCS	10	37.70 ± 7.78	0/10	9.90 ± 6.90	Sham tDCS	BBS, TUG
			38.89 ± 5.71	3.87 ± 1.51		9	37.44 ± 7.89	0/9	9.44 ± 4.30	tDCS	10	40.20 ± 2.43	0/10	9.50 ± 7.32	Core stability exercises	
Akbari et al. (32)	Iran	RCT	42.93 ± 7.95	4.16 ± 0.54	RR	16	42.87 ± 7.99	4/12	NR	Cerebellar tDCS + postural training	14	43.00 ± 8.20	6/8	NR	Sham tDCS + postural training	BBS, TUG
			42.90 ± 8.09	4.09 ± 0.52		16	42.81 ± 8.26	10/6	NR	DLPFC tDCS + postural training	14	43.00 ± 8.20	6/8	NR	Sham tDCS + postural training	
Muñoz-Paredes et al. (33)	Spain	RCT	48.08 ± 8.55	NR	RR SP	12	NR	NR	0.36 ± 0.67	tDCS + physiotherapy	12	NR	NR	0.36 ± 0.67	Sham tDCS + physiotherapy	TUG
Yassine et al. (34)	Egypt	RCT	39.89 ± 6.05	NR	RR	20	39.4 ± 6.3	8/12	3.5 ± 2.8	rTMS + rehabilitation program	19	40.4 ± 5.9	6/13	3.2 ± 3.1	rTMS + rehabilitation program	BBS, TUG, AEs
Rahimibarghani et al. (35)	Iran	RCT	39.91 ± 6.78	4.76 ± 0.81	RR PP SP	21	40.0 ± 7.1	8/13	11.72 ± 3.70	tDCS + aerobic exercise	18	39.8 ± 6.6	7/11	9.32 ± 4.1	Sham tDCS + aerobic exercise	TUG
Cinbaz et al. (36)	Turkey	RCT	48.74 ± 7.82	4.08 ± 0.75	RR PP	11	49 ± 8.75	3/8	16 ± 4.25	tDCS + exercise	12	48.5 ± 7.25	3/9	20 ± 8.75	Sham tDCS + exercise	TUG
			48.75 ± 9.26	4.35 ± 0.95		12	49 ± 11.25	4/8	13.5 ± 8	tsDCS + exercise	12	48.5 ± 7.25	3/9	20 ± 8.75	Sham tDCS + exercise	
Marotta et al. (37)	Italy	RCT	40.6 ± 14.4	3 ± 0.49	RR	9	43.22 ± 10.46	3/6	NR	tDCS + physiotherapy	8	39.75 ± 8.39	2/6	NR	Sham tDCS + physiotherapy	BBS, TUG
Pagliari et al. (38)	Italy	RCT	49.57 ± 10.21	4.50 ± 2.15	NR	20	51.60 ± 8.46	7/13	14.15 ± 9.42	TR RS-AtDCS	20	47.55 ± 11.56	10/10	15.30 ± 10.14	Sham tDCS	TUG, AEs
			51.98 ± 8.91	4.50 ± 1.94		20	51.60 ± 8.46	7/13	14.15 ± 9.42	TR RS-AtDCS	30	52.23 ± 9.34	12/18	15.36 ± 7.17	Usual care	
Pilloni et al. (39)	New York	RCT	53.09 ± 10.74	NR	RR SP	9	52.1 ± 12.85	NR	NR	tDCS + aerobic exercise	8	54.2 ± 8.5	NR	NR	Sham tDCS + aerobic exercise	TUG
Ghosh et al. (40)	Australia	RCT	54 ± 10.9	3.24 ± 1.31	RR PR PP	19	52.21 ± 11.30	6/13	7 ± 4.25	tDCS + physiotherapy	21	55.62 ± 8.82	6/15	12 ± 9	Sham tDCS + physiotherapy	BBS, TUG, AEs
Ehsani et al. (41)	Iran	RCT	35.71 ± 2.51	2.16 ± 0.72	NR	10	35.88 ± 2.21	1/9	NR	tDCS + postural training	10	36.38 ± 2.74	0/10	NR	Sham tDCS + postural training	BBS
Fawaz et al. (42)	Egypt	RCT	35.81 ± 9.12	NR	RR	32	NR	19/13	NR	rTSMS + physiotherapy	32	NR	19/13	NR	Sham rTSMS + physiotherapy	TUG
Nguemeni et al. (43)	Germany	RCT	48.5 ± 9.71	3.77 ± 0.84	NR	12	49.83 ± 10.46	7/5	NR	tDCS	10	46.90 *±* 9.00	3/7	NR	Sham tDCS	TUG

T, treatment group; C, control group; RCT, randomized controlled trial; iTBS, intermittent theta burst stimulation; rTMS, repeated transcranial magnetic stimulation; tDCS, transcranial direct current stimulation; tsDCS, trans-spinal direct current stimulation; TOCT, task-oriented circuit training; DLPFC, dorsolateral prefrontal cortex; TR RS-AtDCS, telerehabilitation with active transcranial direct current stimulation; rTSMS, repetitive trans-spinal magnetic stimulation; M1, primary motor cortex; PP, primary progressive; RR, relapsing remitting; SP, secondary progressive; PR, progressive-relapsing; TUG, Timed Up and Go; BBS, Berg Balance Scale; AEs, adverse events; NR, not reported.

**Table 2:** Intervention details of the included studies.

Study	Target electrode location	Intensity	Frequency	Treatment duration	Adverse event
Tramontano et al. (27)	Bilateral cerebellar hemispheres	NR	40 min/per time, 5 times/week	2 weeks	/
Mohamed et al. (28)	Bilateral cerebellar hemispheres	NR	20 min/per time, 3 times/week	2 weeks	Headache
Charehjou et al. (29)	+: Primary motor cortex (M1, C3) −: Right forehead	2 mA	20 min/per time, 3 times/week	2 weeks	/
Baroni et al. (30)	+: Cerebellum right hemisphere −: Ipsilateral bucciNRtor	2 mA	2 h/per time, 5 times/week	2 weeks	Tingling, skin redness, headache, trouble to concentrate, sleepiness, pain in the site of stimulation, mood fluctuations
Mohammadkhanbeig et al. (31)	+: Primary motor cortex (M1, Cz) −: Supraorbital area	2 mA	20 min/per time, 5 times/week	6 weeks	/
Akbari et al. (32)	+: Cerebellum (1 cm below inion of occipital bone) −: Right bucciNRtor muscle	1.5 mA	20 min/per time, a 48-h interval between sessions	4 weeks	/
	+: Left dorsolateral prefrontal cortex −: Right supraorbital region				/
Muñoz-Paredes et al. (33)	+: Left DLPFC −: Right supraorbital cortex	2 mA	20 min/per time, a total of 10 sessions	4 weeks	/
Yassine et al. (34)	Bilateral cerebellar hemispheres	NR	20 min/per time, a total of 12 sessions	4 weeks	Nauseous, headache, dizziness
Rahimibarghani et al. (35)	+: Primary motor cortex (M1, C3) −: Contralateral shoulder	1.5 mA	20 min/per time, 2 times/week	6 weeks	/
Cinbaz et al. (36)	+: Primary motor cortex (M1, C3) −: Supraorbital region	2 mA	20 min/per time, 3 times/week	4 weeks	/
	+: T10 spinous process −: Left posterior deltoid				/
Marotta et al. (37)	+: Primary motor cortex (M1, C3) −: Fp2 (supraorbital margin)	2 mA	20 min/per time, 5 times/week	2 weeks	/
Pagliari et al. (38)	+: Left DLPFC −: Right DLPFC	2 mA	45 min/per time with addition NRl 20 min of tDCS during the first week, 5 times/week	6 weeks	Skin irritation, pain, burning sensation, heat sensation, itching, iron taste, fatigue
Pilloni et al. (39)	+: Primary motor cortex (M1, C3) −: Fp2 (supraorbital margin)	2.5 mA	20 min/per time	NR	/
Ghosh et al. (40)	+: Primary motor cortex (M1, Cz) −: Supraorbital area	2 mA	20 min/per time, 2 times/week	6 weeks	Tingling, itching, burning, scalp pain, sleepiness, trouble concentrating
Ehsani et al. (41)	+: Cerebellum right hemisphere −: Right bucciNRtor muscle	1.5 mA	20 min/per time, 5 times/week	2 weeks	/
Fawaz et al. (42)	C7 cervical vertebrae	2 mA	5 s/per time, 10-s intertrain interval, 30 trains in total. 5 times/week	2 weeks	/
Nguemeni et al. (43)	+: Cerebellum (3 cm lateral to the inion) −: Ipsilateral bucciNRtor muscle	2 mA	15 min/per time, 3 times/week	2 weeks	/

C3 and Cz are electrode positions according to the international 10–20 EEG system, corresponding to the left primary motor cortex and midline motor cortex, respectively.

### 3.3 Quality assessment of included studies

**Figure 2:**
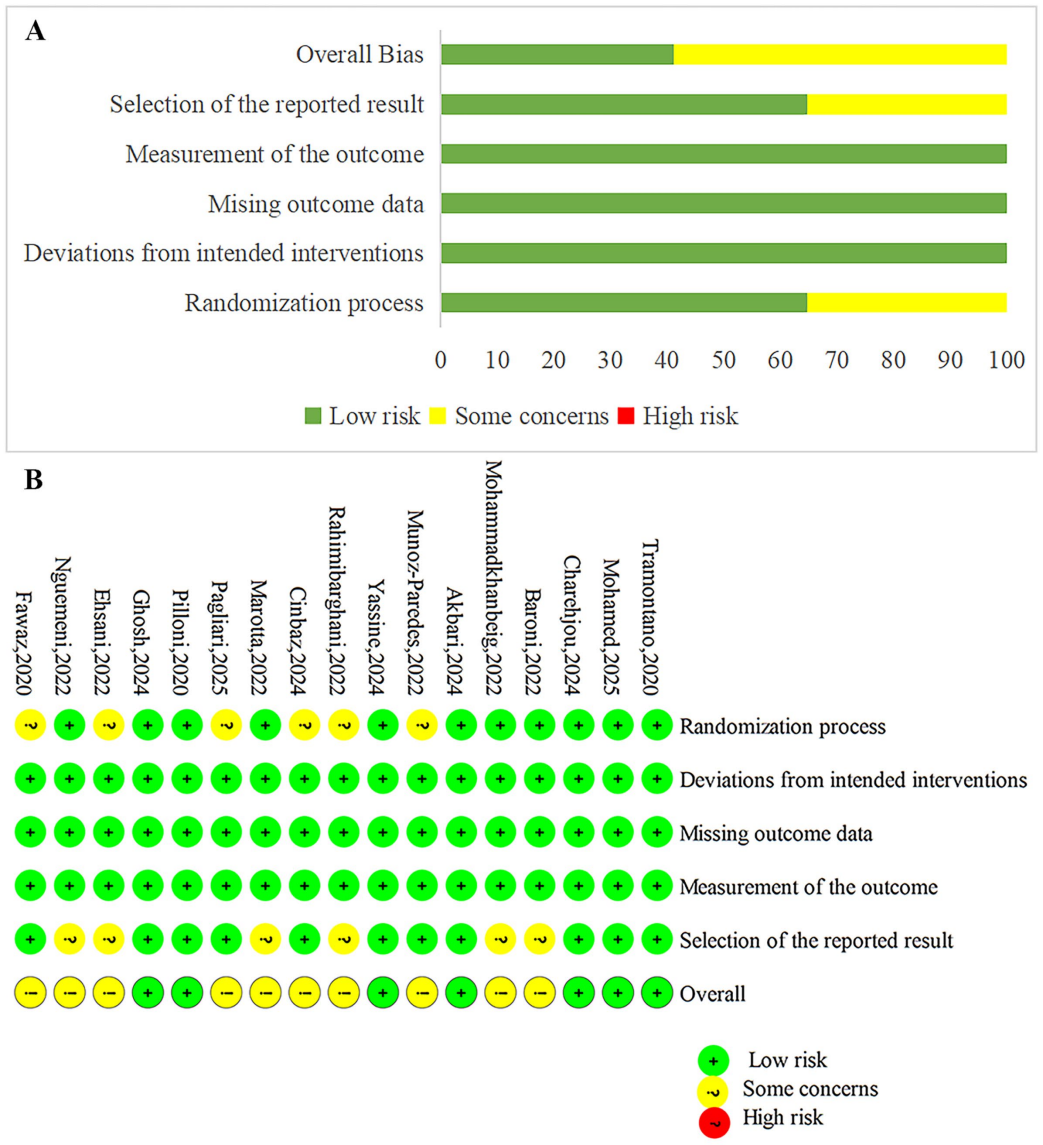
Risk of bias summary and graph. **(A)** The risk of bias profile across. **(B)** The detailed results of the risk of bias.

The quality of the 17 included studies was assessed using the Cochrane Risk of Bias tool. Fifteen described random sequence generation, 11 reported allocation concealment, and 9 implemented blinding of both participants and assessors. All studies provided complete data reporting and explained their outcomes. Overall, the methodological quality of the included studies was relatively high. The quality evaluation of the included literature is shown in Figure 2.

### 3.4 Meta-analysis results

#### 3.4.1 Overall results

**Figure 3:**
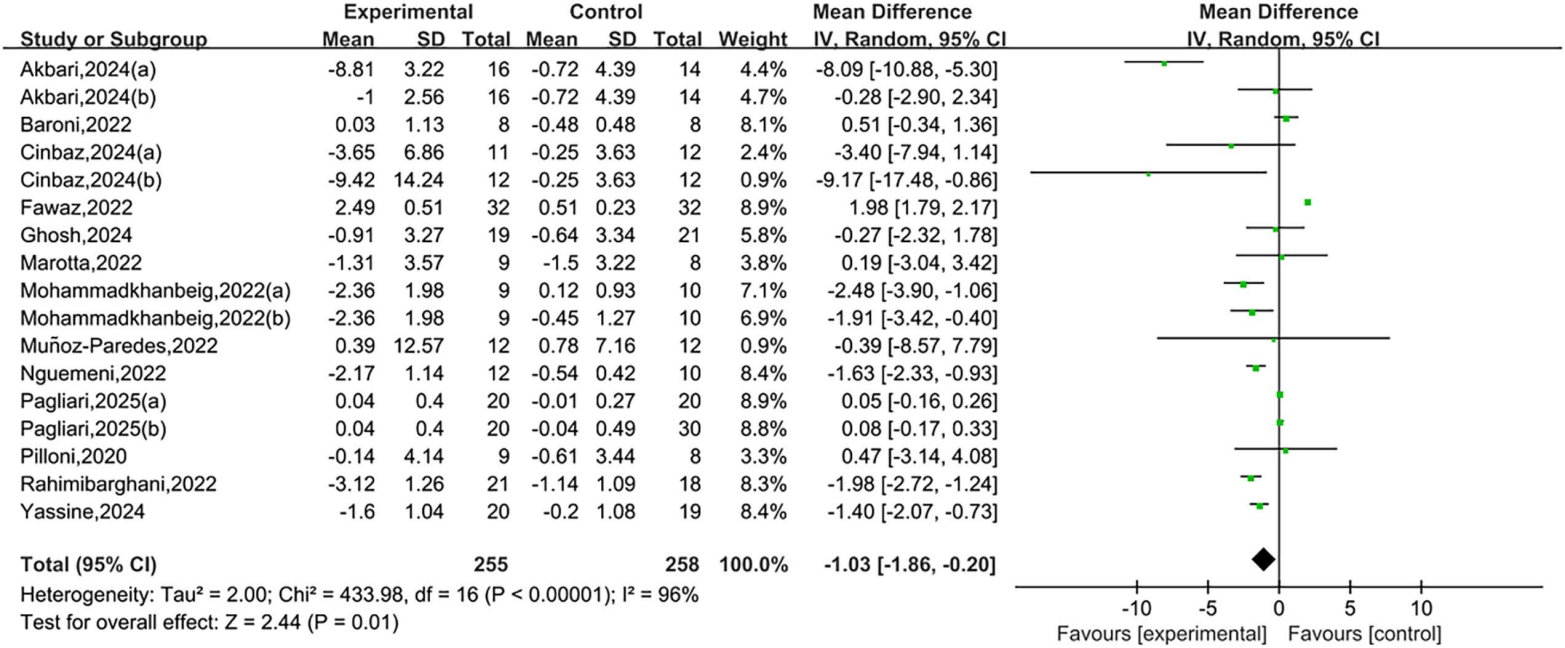
Forest plots of TUG.

A total of 13 studies reported TUG results. Heterogeneity testing showed *I*^2^ = 96%, *p* < 0.00001, indicating substantial heterogeneity between studies. A random-effects model was used for the analysis. Meta-analysis showed that NIBS group had significantly shorter than that in the control group, and the difference was statistically significant [MD = −1.03, 95% CI (−1.86, −0.20), *p* = 0.01], as shown in Figure 3. A negative mean difference indicates a greater reduction in TUG completion time in the NIBS group, suggesting improved balance ability.

**Figure 4:**
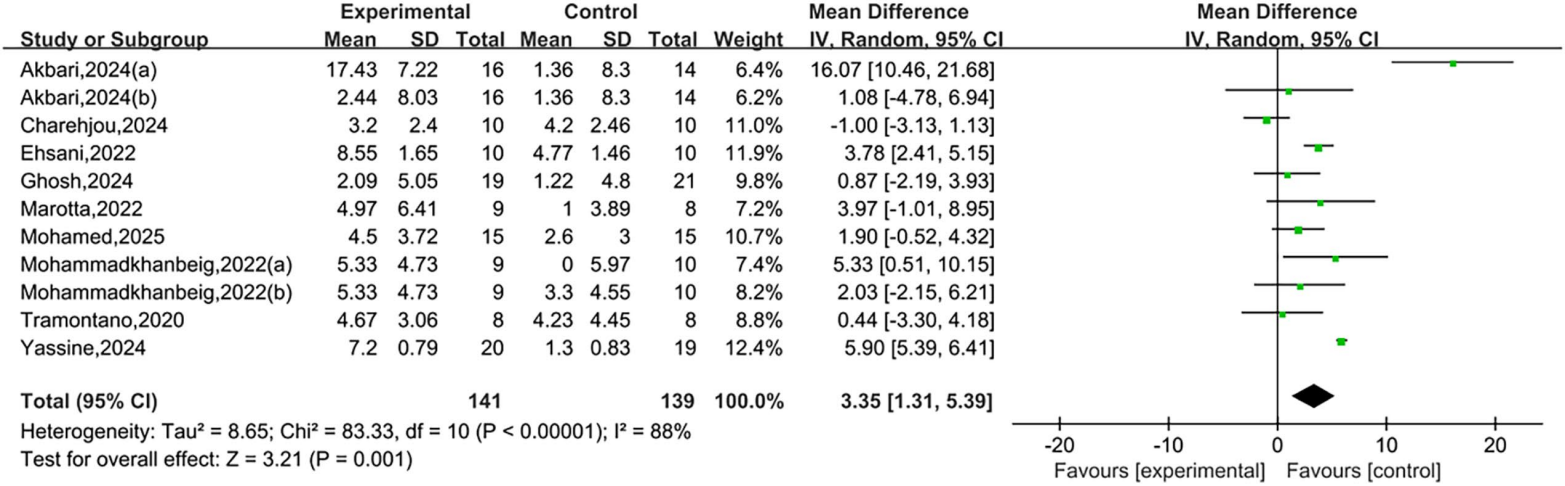
Forest plots of BBS.

A total of nine studies reported BBS. Heterogeneity testing showed *I*^2^ = 88%, *p* < 0.00001, indicating substantial heterogeneity between studies. A random-effects model was used for analysis. Meta-analysis results showed that NIBS group had significantly higher BBS scores than the control group, with a statistically significant difference [MD = 3.35, 95% CI (1.31, 5.39), *p* = 0.001], as shown in Figure 4. A positive mean difference indicates that the NIBS group had higher BBS scores, suggesting better balance ability and a lower risk of falls.

**Figure 5:**
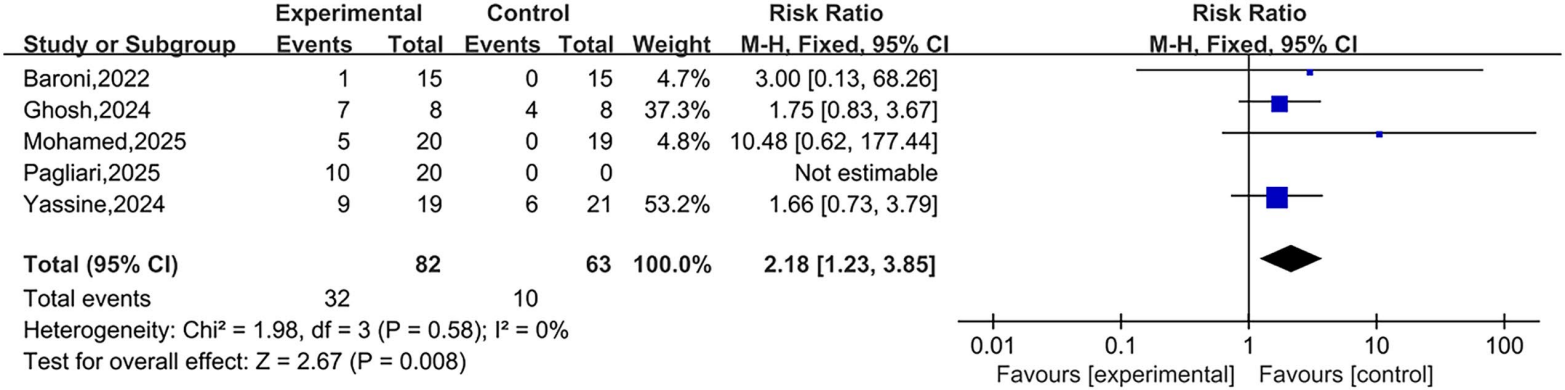
Forest plots of AEs.

A total of five studies reported the occurrence of AEs. Heterogeneity testing showed *I*^2^ = 0%, *p* = 0.58, indicating low heterogeneity between the studies. A fixed-effect model was used for the pooled analysis. Meta-analysis results indicated that the incidence of AEs in NIBS group was higher than that in control group, with the difference being statistically significant [RR = 2.18, 95% CI (1.23, 3.85), *p* = 0.008]. It is noteworthy that the vast majority of reported AEs were mild, transient, and self-limiting, typically resolving shortly during or after the stimulation without the need for special intervention. Therefore, this result does not suggest that NIBS is a high-risk intervention, but rather objectively confirms the statistical significance of its known and commonly observed mild side effects. The forest plot is presented in Figure 5.

#### 3.4.2 Subgroup analysis

Due to the high heterogeneity between studies, subgroup analyses were conducted to further explore potential sources of heterogeneity. Subgroup analysis of the TUG showed that among different NIBS types, tDCS reduced TUG time [MD = −1.14, 95% CI (−1.80, −0.49)], while rTMS showed no significant improvement. Regarding MS subtypes, patients with relapsing remitting (RR) MS showed a trend toward improvement [MD = −1.61, 95% CI [−3.77, 0.55]], although the difference did not reach statistical significance. Other mixed subtypes also did not demonstrate significant differences. This suggests that tDCS may be a more effective NIBS technique, and its efficacy may be associated with the MS subtype. However, given the limited number of studies within certain subgroups, these findings should be interpreted with caution. Variations in intervention duration (*p* = 0.13, *I*^2^ = 51.7%) and stimulation intensity (*p* = 0.12, *I*^2^ = 58.3%) were not statistically significant contributors to outcome differences. However, within-group analysis of intervention durations indicated that a 4-week intervention was more effective [MD = −3.42, 95% CI (−6.32, −0.53)], and a 6-week intervention also showed significant improvement [MD = −0.88, 95% CI (−1.55, −0.21)], while a 2-week intervention did not yield statistically significant benefits. These results suggest that a minimum of 4 weeks of NIBS intervention may be required to achieve meaningful improvements in balance function in MS patients. Therefore, these factors are unlikely to account for the observed heterogeneity. Subgroup analysis by stimulation site showed no significant differences in the cerebellum and left dorsolateral prefrontal cortex (DLPFC) groups. Stimulation targeting the primary motor cortex (M1) showed positive effects, particularly in the M1 (Cz) region [MD = −1.74, 95% CI (−2.90, −0.58)]. Due to the limited number of studies in each subgroup, the results may be unstable; however, stimulation site is likely a potential source of heterogeneity. See Table 3 and Supplementary Figures S1–S5.

**Table 3:** Subgroup analysis results of TUG.

Category	Subgroup	Number of included studies (*n*)	Heterogeneity test result		Effect model	Meta-analysis result		
			*I*^2^ (%)	*p*		MD	95% CI	*p*
MS subtype	RR	7	97	<0.00001	Random	−1.61	[−3.77, 0.55]	0.14
	RR + PP + SP	3	90	<0.0001		−0.62	[−2.49, 1.26]	0.52
	RR + PP	2	30	0.23		−5.19	[−10.42, 0.04]	0.05
	RR + SP	2	0	0.85		0.33	[−2.97, 3.63]	0.84
NIBS types	tDCS	15	86	<0.00001	Random	−1.14	[−1.80, −0.49]	0.0006
	rTMS	2	99	<0.00001		0.31	[−3.01, 3.62]	0.86
Intervention duration	2 weeks	4	97	<0.00001	Random	0.28	[−1.83, 2.39]	0.79
	4 weeks	6	81	<0.0001		−3.42	[−6.32, −0.53]	0.02
	6 weeks	6	89	<0.00001		−0.88	[−1.55, −0.21]	0.01
Stimulation intensity	1.5 mA	3	90	<0.0001	Random	−3.34	[−7.00, 0.32]	0.07
	2 mA	11	97	<0.00001		−0.35	[−1.29, 0.58]	0.46
Stimulation site	Cerebellum	2	95	<0.0001	Random	−4.71	[−11.04, 1.61]	0.14
	Left DLPFC	4	0	0.99		0.06	[−0.10, 0.22]	0.46
	M1 (C3)	4	19	0.3		−1.54	[−2.79, −0.30]	0.01
	M1 (Cz)	3	34	0.22		−1.74	[−2.90, 0.58]	0.003

A total of 17 RCTs were included in this study; however, 3 of them contained multiple intervention groups, which were treated as independent comparisons in this subgroup analysis. The number of studies listed for each subgroup refers to the number of independent comparisons.

Subgroup analysis of BBS showed that both tDCS [MD = 3.57, 95% CI (0.84, 6.31)] and rTMS [MD = 4.08, 95% CI (0.18, 7.99)] were associated with improvements in BBS scores, suggesting that different types of NIBS may be effective in enhancing static balance function. Patients with RR MS demonstrated greater improvement [MD = 5.61, 95% CI (2.57, 8.65)], whereas those with RR + SP (secondary progressive) MS did not show significant benefits (*p* = 0.16). However, neither NIBS type nor MS subtype appeared to be significant sources of heterogeneity in BBS. No statistically significant differences were observed between subgroups based on intervention duration (*p* = 0.26, *I*^2^ = 26.5%), stimulation intensity (*p* = 0.21, *I*^2^ = 37.6%), or stimulation site (*p* = 0.79, *I*^2^ = 0%). Nevertheless, within-group analysis revealed that a 4-week intervention was particularly effective [MD = 7.57, 95% CI (0.99, 14.15)]. Stimulation targeting M1 (Cz) showed low heterogeneity (*I*^2^ = 15%), indicating that stimulation at other sites may be potential sources of inter-study heterogeneity. For further details, see Table 4 and Supplementary Figures S6–S10.

**Table 4:** Subgroup analysis results of BBS.

Category	Subgroup	Number of included studies (*n*)	Heterogeneity test result		Effect model	Meta-analysis result		
			*I*^2^ (%)	*p*		MD	95% CI	*p*
MS subtype	RR	6	74	0.002	Random	5.61	[2.57, 8.65]	0.0003
	RR + SP	2	0	0.52		1.47	[−0.56, 3.50]	0.16
NIBS types	tDCS	8	82	<0.00001	Random	3.57	[0.84, 6.31]	0.01
	rTMS	2	90	0.002		4.08	[0.18, 7.99]	0.04
Intervention duration	2 weeks	5	74	0.004	Random	1.74	[−0.48, 3.95]	0.12
	4 weeks	3	87	0.0005		7.57	[0.99, 14.15]	0.02
	6 weeks	3	15	0.31		2.21	[−0.21, 4.63]	0.07
Stimulation intensity	1.5 mA	3	89	<0.0001	Random	6.81	[−0.70, 14.32]	0.08
	2 mA	3	61	0.05		1.63	[−1.16, 4.43]	0.25
Stimulation site	M1 (C3)	2	69	0.07	Random	0.95	[−3.80, 5.71]	0.69
	M1 (Cz)	3	15	0.31		2.21	[−0.21, 4.63]	0.07
	Bilateral cerebellar hemispheres	3	89	0.0002		3.05	[−0.59, 6.69]	0.1

#### 3.4.3 Meta-regression

To investigate potential sources of the observed heterogeneity, meta-regression analyses were performed separately for TUG and BBS using the restricted maximum likelihood (REML) method, based on clinically relevant covariates. The included covariates were mean patient age and baseline EDSS score.

**Figure 6:**
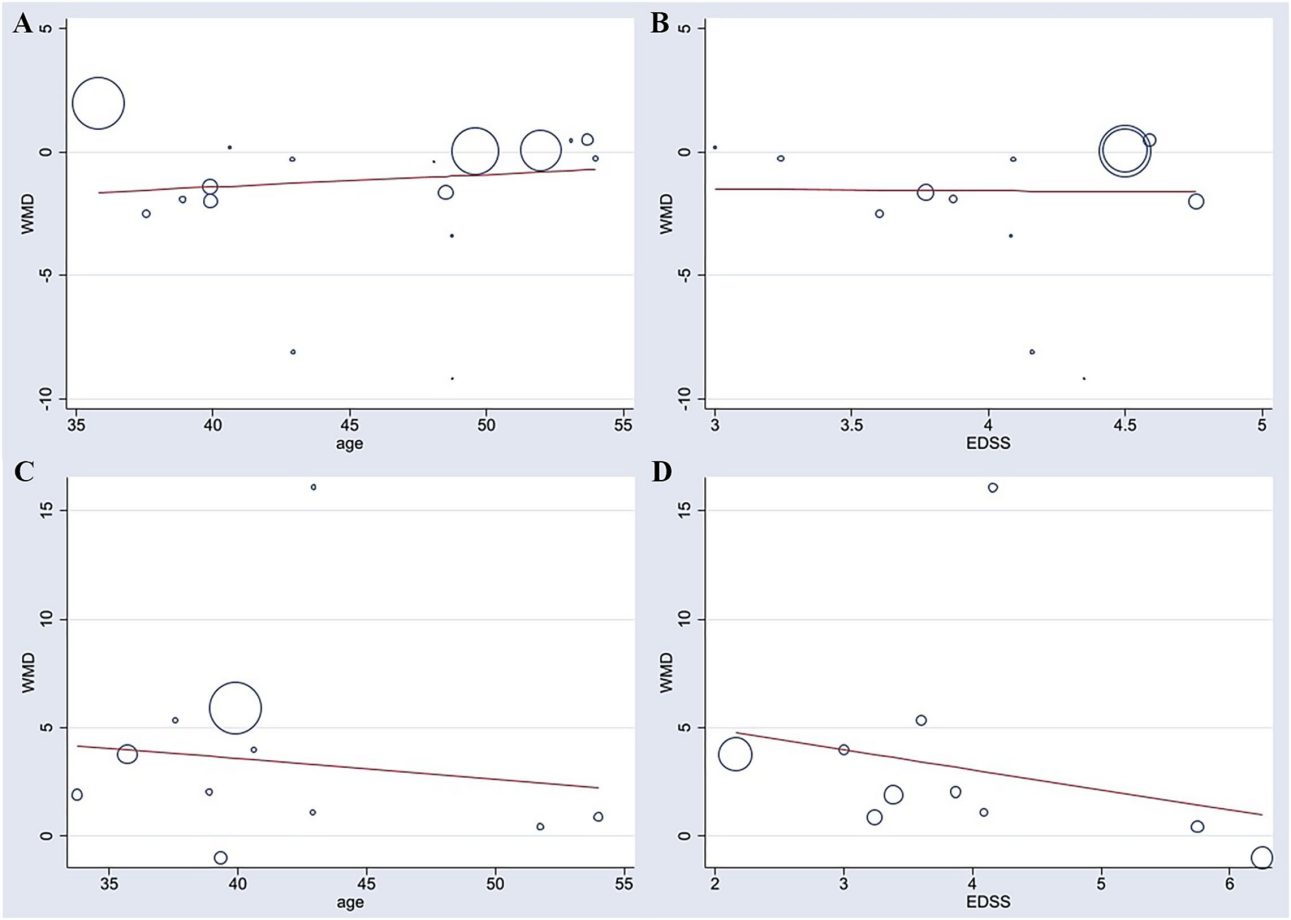
Meta-regression analyses of patient age and baseline EDSS score with improvements in TUG **(A,B)** and BBS **(C,D)**.

For TUG, a total of 13 studies were included in the meta-regression. The results indicated that neither age (*β* = 0.154, *p* = 0.214) nor EDSS score (*β* = −0.308, *p* = 0.821) was a significant predictor of the effect size. The overall model demonstrated low explanatory power, with an adjusted R^2^ of only 12.53%, and was not statistically significant [*F* (2, 10) = 0.88, *p* = 0.444]. After including these two covariates, substantial residual heterogeneity remained (*I*^2^_res = 76.51%, *τ*^2^ = 3.129). Therefore, this meta-regression did not identify age or EDSS score as significant contributors to the heterogeneity observed in the TUG meta-analysis (Figure 6).

For BBS, a total of 10 studies were included in the meta-regression. Similarly, age (*β* = −0.034, *p* = 0.897) and EDSS score (*β* = −0.875, *p* = 0.534) were not significant predictors of the effect size. The model showed poor explanatory power, with an adjusted *R*^2^ of −34.12%, and was not statistically significant [*F* (2, 7) = 0.27, *p* = 0.769]. Substantial residual heterogeneity remained after adjusting for the covariates (*I*^2^_res = 75.56%, *τ*^2^ = 18.06). These findings suggest that the heterogeneity may be attributable to other unknown study-level characteristics (Figure 6).

### 3.5 Sensitivity analysis

**Figure 7:**
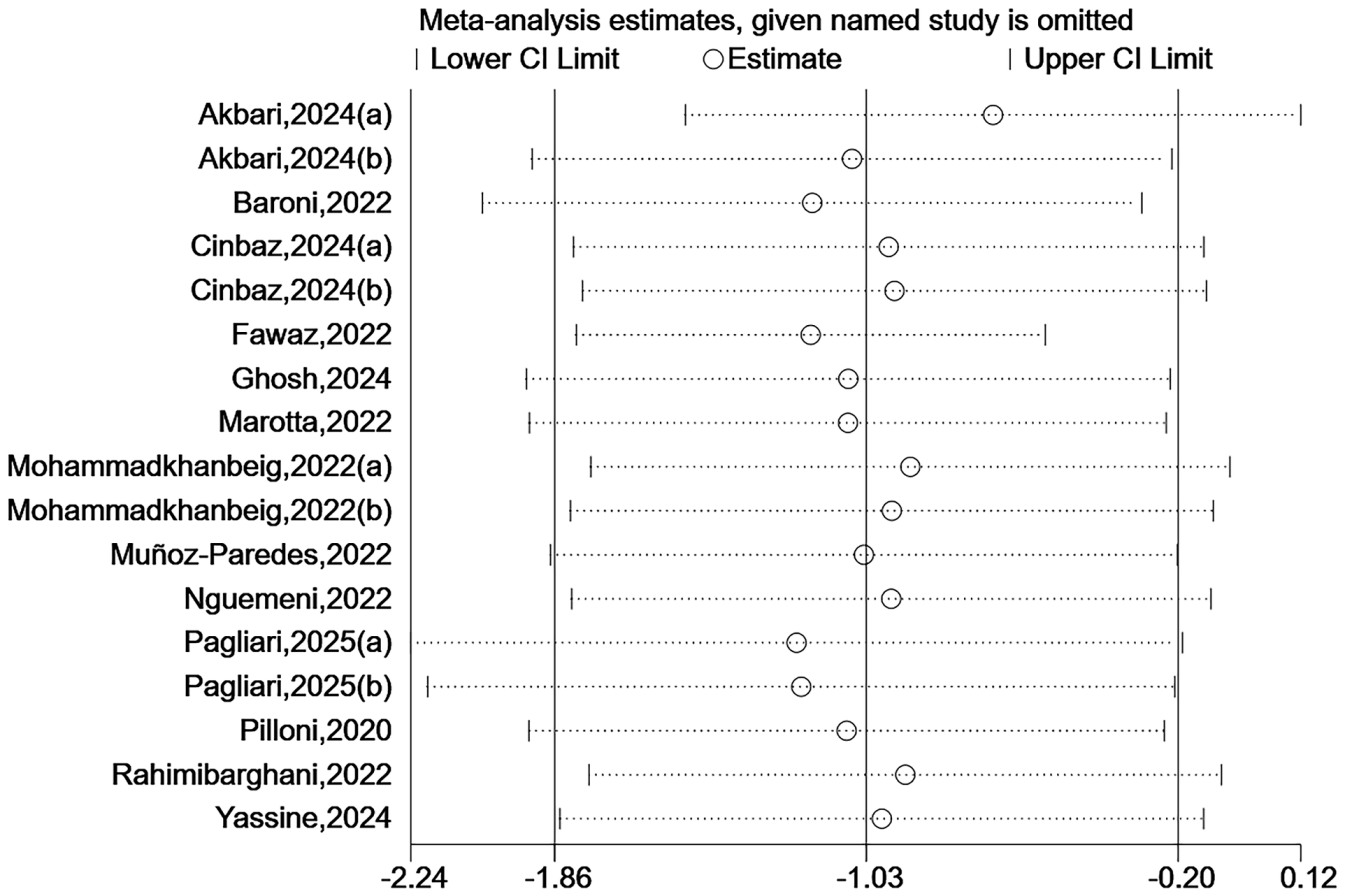
Sensitivity analysis for TUG.

**Figure 8:**
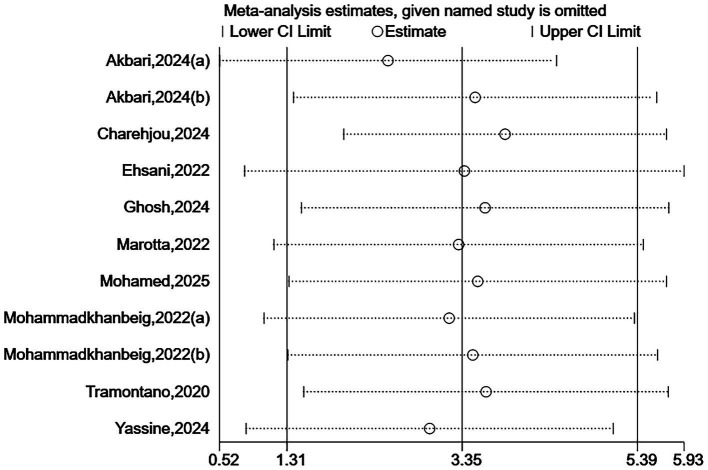
Sensitivity analysis for BBS.

Due to some heterogeneity between studies, we sequentially excluded each study to assess the stability and reliability of the combined results. The results showed that the combined effect size did not change direction, indicating that despite the high heterogeneity among the included studies, the overall findings were robust. See Figures 7, 8.

### 3.6 Publication bias

**Figure 9:**
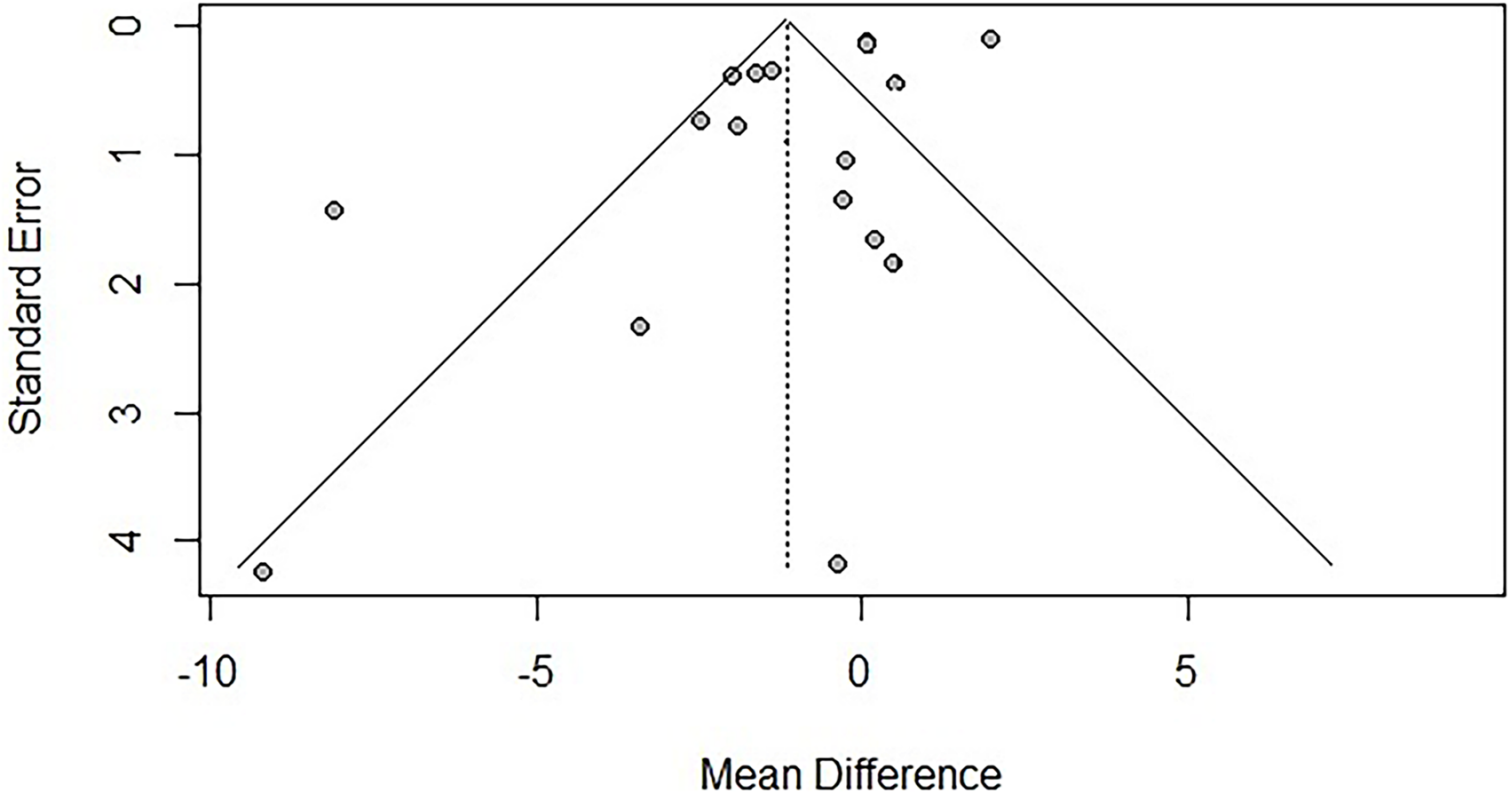
Funnel plot for TUG.

**Figure 10:**
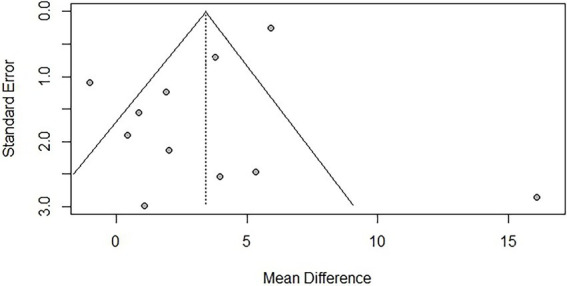
Funnel plot for BBS.

To assess publication bias, funnel plots were created, and Egger’s test was used to evaluate the symmetry of the funnel plots. The results showed that there was publication bias for TUG (*p* = 0.041 < 0.05), suggesting that negative results may not have been published. However, trim-and-fill analysis [−1.144, 95% CI (−2.203, −0.005)] indicated that no additional studies were needed to make the funnel plot symmetrical, suggesting that the impact of publication bias on the overall results for TUG was minimal. No significant publication bias was found for BBS (*p* = 0.121). Due to the limited number of studies reporting AEs (*n* < 10), publication bias was not assessed. The funnel plots are shown in Figures 9, 10.

### 3.7 Quality of evidence assessment

The quality of evidence for the primary outcomes, TUG and BBS, was rated as moderate certainty. The downgrading was due to the risk of bias in the included studies (e.g., inadequate randomization and blinding in some trials) and substantial heterogeneity (*I*^2^ > 80%). However, the direction of effect was consistent across studies, and the results were robust.

The quality of evidence for AEs was rated as low certainty, primarily due to inconsistencies in the definition and reporting of adverse events across studies, as well as imprecision, reflected by wide confidence intervals and limited sample sizes. See Table 5 for details.

**Table 5:** Assessment of quality of evidence.

Outcome	Participants (studies)	Risk of bias	Inconsistency	Indirectness	Imprecision	Publication bias	*I*^2^ (%)	95% CI	Certainty (GRADE)
TUG	448 (13 RCTs)	Serious ①	Serious ②	Not serious	Not serious	Possible	96	[−1.86, −0.20]	⨁ ⨁ ⨁ ◯ Moderate
BBS	257 (9 RCTs)	Serious ①	Serious ②	Not serious	Not serious	Unlikely	88	[1.31, 5.39]	⨁ ⨁ ⨁ ◯ Moderate
AEs	195 (5 RCTs)	Serious ③	Not serious	Not serious	Serious ④	Possible	0	[1.23, 3.85]	⨁ ⨁ ◯ ◯ Low

① Some studies had a “some concerns” risk in terms of randomization and blinding procedures. ② There was substantial statistical heterogeneity across studies (*I*^2^ > 50%). ③ Definitions, monitoring methods, and reporting of adverse events varied greatly among studies, with incomplete reporting. ④ Wide confidence intervals and a limited number of studies with small sample sizes contributed to imprecision in the results.

## 4 Discussion

This study conducted a systematic review and meta-analysis to comprehensively evaluate the efficacy and safety of NIBS in improving balance function in patients with MS, providing relatively robust evidence to support clinical practice. The results show that NIBS has shown certain improvements in the balance abilities of MS patients, specifically reflected in the following aspects:

1. Meta-analysis revealed an association between NIBS intervention and a reduction in TUG completion time, suggesting that NIBS may help improve patients’ dynamic balance. In parallel, the increase in BBS scores indicated a potential improvement in static balance function.
2. Among different types of NIBS techniques, tDCS demonstrated consistent benefits for both dynamic and static balance, whereas the evidence supporting the efficacy of rTMS was relatively limited. In terms of MS subtypes, patients with RR MS appeared to benefit more from NIBS intervention, particularly with regard to improvements in static balance.
3. Subgroup analysis of intervention parameters indicated that both 4-week and 6-week interventions were associated with reductions in TUG time, and a 4-week intervention may also contribute to improvements in BBS. Regarding stimulation sites, targeting the M1, specifically the C3 and Cz regions, yielded relatively better outcomes, whereas stimulation of the cerebellum and prefrontal cortex did not show significant benefits. No statistically significant differences were observed across different NIBS stimulation intensities.
4. NIBS interventions were associated with AEs such as itching, warmth, burning sensations, and fatigue. However, most of these reactions were mild and transient. Since the control group did not receive electrical stimulation, adverse events were rarely reported in that group. These findings highlight the need to carefully weigh the therapeutic benefits of NIBS against its safety profile in clinical applications, and to enhance active monitoring and standardized reporting of AEs.

These improvements may stem from the multifaceted positive effects of NIBS on balance function in patients with MS. Individuals with MS often exhibit reduced excitability in M1 and impaired corticospinal tract conduction. Anodal tDCS or high-frequency rTMS can modulate neuronal membrane potentials, thereby enhancing M1 excitability and reversing pathological hypoexcitability (44, 45). Through this mechanism, NIBS strengthens motor commands from the brain to the spinal cord, improving motor output efficiency and muscle control precision, which in turn facilitates faster and more accurate postural adjustments (45). In addition, NIBS is believed to modulate the functional balance of distributed neural networks and enhance connectivity among different brain regions, such as the motor cortex, cerebellum, and somatosensory cortex (46, 47). This reorganization of neural networks may help compensate for MS-related neural pathway damage, optimizing the integration of sensory information and the generation of motor commands—both of which are fundamental to maintaining balance (46). It is worth noting that most of the included studies combined NIBS with conventional rehabilitation therapies (e.g., balance training, aerobic exercise). Therefore, NIBS may enhance cortical plasticity during rehabilitation, making concurrent motor learning more effective and thereby reinforcing and consolidating the effects of therapeutic interventions.

Furthermore, the heterogeneity in treatment response observed in our subgroup analyses may also be attributed to the intrinsic variability of MS itself, including phenotypic diversity and baseline disability levels. Patients with RRMS and lower EDSS scores typically retain greater neural plasticity and functional reserve capacity, potentially rendering them more responsive to neuromodulatory interventions like NIBS. Conversely, those with progressive forms (PPMS, SPMS) and higher EDSS scores often exhibit more extensive neurodegenerative changes and reduced brain reserve, which might constrain their capacity for functional reorganization following stimulation (48, 49). These considerations are supported by Iodice et al. (50), which systematically elaborated on how MS phenotypic heterogeneity and disability severity are critical determinants of rehabilitation outcomes, underscoring the necessity for individualized and stratified therapeutic approaches in this population. This underscores the importance of considering MS subtype and disability severity as key modifiers of NIBS effects, which should be carefully stratified in future clinical trials to identify the patient subgroups most likely to benefit.

The stimulation site is a critical factor influencing the efficacy of NIBS. M1 as the final common pathway for motor output, directly controls lower limb muscle contractions (51). Therefore, stimulation targeting the M1 region may more directly optimize the generation of motor commands required for balance control. In the international 10–20 EEG system, the central point (Cz) approximately corresponds to the cortical representation area of the lower limbs, making it a potentially more precise and targeted stimulation site (52). In contrast, cerebellar stimulation did not show consistent advantages in this meta-analysis. Several factors may account for this finding. First, due to the cerebellum’s deep anatomical location, there is ongoing debate over whether transcranial electrical stimulation can effectively penetrate the skull and reach the cerebellar cortex with sufficient field intensity (53). Second, the cerebellum is a commonly affected region in MS, and inherent structural damage, such as white and gray matter atrophy may disrupt functional connectivity. This disconnection could reduce the cerebellum’s responsiveness to neuromodulatory interventions such as TMS or tDCS (54). Third, the cerebellum primarily contributes to motor coordination, timing, prediction, and motor learning, rather than initiating movement or generating muscular force directly (55).

In terms of stimulation parameters, although this study did not identify a significant effect of different stimulation intensities on balance improvement in MS, subgroup analysis of intervention duration yielded valuable insights: both 4-week and 6-week interventions were associated with reductions in TUG completion time, and the 4-week intervention also appeared to contribute to improvements in BBS. These findings suggest that the therapeutic effect of NIBS may stabilize once certain threshold parameters are met, and further increases in stimulation intensity or total treatment duration may not produce additional benefits. This phenomenon may be related to the biological characteristics of tDCS, whose ultimate effects are likely the result of complex interactions among multiple dose-related parameters, including current intensity, stimulation duration, electrode size and configuration, and the number of treatment sessions (56). The present findings are consistent with those of Emadi et al. (21), who reported that single-session stimulation is typically insufficient to induce sustained plastic changes. Instead, repeated sessions, such as those delivered over at least 4 weeks in the current study are crucial for achieving cumulative effects and long-term benefits. Therefore, ensuring an adequate intervention duration may be a key factor in eliciting clinically measurable improvements.

This study found that the incidence of adverse events was significantly higher in the NIBS intervention group compared to the control group. However, the vast majority of reported adverse events were mild and transient, such as skin tingling, itching at the stimulation site, mild headache, and a burning sensation. Serious adverse events were rarely observed. These discomforts may be related to factors such as locally elevated current density, the quality of the conductive medium, the method of current ramp-up/ramp-down, or individual differences in skin sensitivity (56, 57). Despite the increased risk of adverse events, their mild and temporary nature suggests that the overall safety profile of NIBS remains favorable.

Compared with previous studies, the study by Nombela-Cabrera et al. (20) focused solely on tDCS and found improvements in gait function, while improvements in static balance did not reach statistical significance. Additionally, no significant differences were observed when grouping by stimulation site. In contrast, the present study included a broader range of NIBS techniques (including rTMS, iTBS, etc.) and a larger number of studies, confirming that NIBS can improve both dynamic and static balance. This discrepancy may be attributed to the broader inclusion criteria and larger sample size in the present analysis, suggesting that different types of NIBS may exert synergistic effects through shared neuromodulatory mechanisms, rather than tDCS being the only effective modality. Emadi et al. (21) primarily investigated spasticity and gait speed, reporting that multi-session TMS effectively improved spasticity, while single-session tDCS had no significant impact on gait speed. However, their study did not analyze balance-specific outcomes, limiting the strength of its conclusions regarding balance function. In contrast, our study not only focused on key indicators of balance function but also assessed the safety of NIBS in this population by reporting the risk of adverse events—an aspect of high practical relevance for clinical decision-making. Furthermore, through subgroup analyses and meta-regression, we explored the potential impact of various factors such as stimulation targets, stimulation intensity, intervention parameters, and patients’ baseline characteristics on treatment efficacy. These analyses enhanced the interpretability of the results and provided important evidence to guide the individualization and precision of treatment strategies in future clinical practice.

Despite the comprehensive literature search and rigorous methodology employed in this meta-analysis, several limitations should be acknowledged. First, our search was restricted to articles published in English, which may have introduced language bias and led to the omission of potentially relevant studies published in other languages. Second, although subgroup analyses and meta-regression were conducted to explore the heterogeneity and stimulation parameters reported in previous studies, substantial residual heterogeneity remained. This may be due to variations in study design, types of concurrent rehabilitation programs, and stimulation parameters (e.g., session duration, electrode size, or coil type) across the included trials. The conclusions drawn from certain subgroups (e.g., cerebellar stimulation) require further validation through future studies. As highlighted by Iodice et al. (58) in their comprehensive review, this variability represents a fundamental challenge in the NIBS field for MS and may account for the inconsistent findings across studies. The analysis of safety outcomes was limited by the incomplete and inconsistent reporting of adverse events in the original studies. Only a small number of trials reported adverse events, and definitions, data collection methods, and reporting standards varied significantly. This inconsistency may introduce bias in the assessment of NIBS safety. Future research should adopt standardized adverse event reporting guidelines, such as the CONSORT extension for harms, and enhance monitoring and documentation during interventions. Standardizing the reporting process will allow for a more comprehensive and accurate evaluation of the safety profile of NIBS. Lastly, with respect to long-term outcomes, most of the included studies had relatively short follow-up periods and primarily assessed immediate or short-term effects. As a result, this meta-analysis cannot draw definitive conclusions regarding the durability of NIBS effects over time. Future research should prioritize well-designed, large-scale randomized controlled trials with long-term follow-up (e.g., at 3, 6, and 12 months post-intervention), focusing on optimizing strategies for sustained intervention effects during the maintenance phase.

## 5 Conclusion

As an adjunctive therapeutic approach, NIBS has shown certain beneficial effects in improving balance control in patients with MS. It provides a non-pharmacological, non-invasive treatment option for the rehabilitation of balance dysfunction in MS, particularly suitable for patients who are unresponsive to or intolerant of pharmacological therapies. However, the potential for mild adverse effects should not be overlooked. Future studies should prioritize large-scale, high-quality randomized controlled trials focusing on standardized and targeted stimulation protocols, long-term follow-up to assess the sustainability of effects, and comprehensive, standardized reporting of adverse events. These efforts are essential to validate the findings of this study, optimize stimulation parameters, and facilitate the integration of NIBS into individualized rehabilitation strategies for MS.

## Data Availability

The original contributions presented in the study are included in the article/Supplementary material, further inquiries can be directed to the corresponding author.
