# Supplementary material for: Effects of non-invasive brain stimulation on balance control in patients with multiple sclerosis: a systematic review and meta-analysis
Source: Front Neurol. 2025 Oct 10;16:1696343. doi: 10.3389/fneur.2025.1696343 (PMC12549307; doi:10.3389/fneur.2025.1696343)

Supplementary material

Effects of Non-Invasive Brain Stimulation on Balance Control in Patients with Multiple Sclerosis: A Systematic Review and Meta-Analysis

Table of contents

Table S1: PRISMA NMA Checklist 2
Table S2: Search strategy 6
Figure S1:Forest plot of subgroup analysis based on NIBS type of TUG. 9
Figure S2:Forest plot of subgroup analysis based on MS subtype of TUG. 9
Figure S3:Forest plot for subgroup analysis based on intervention duration of TUG. 10
Figure S4:Forest plot for subgroup analysis based on stimulation intensity of TUG. 10
Figure S5:Forest plot for subgroup analysis based on stimulation site of TUG. 11
Figure S6:Forest plot of subgroup analysis based on NIBS type of BBS. 11
Figure S7:Forest plot of subgroup analysis based on MS subtype of BBS. 11
Figure S8:Forest plot for subgroup analysis based on intervention duration of BBS. 12
Figure S9:Forest plot for subgroup analysis based on stimulation intensity of BBS. 12
Figure S10:Forest plot for subgroup analysis based on stimulation site of BBS. 12

Table S1: PRISMA NMA Checklist

Section and Topic	Item #	Checklist item	Location where item is reported
**TITLE**			
Title	1	Identify the report as a systematic review.	Title
**ABSTRACT**			
Abstract	2	See the PRISMA 2020 for Abstracts checklist.	Abstract
**INTRODUCTION**			
Rationale	3	Describe the rationale for the review in the context of existing knowledge.	Introduction, paragraph three
Objectives	4	Provide an explicit statement of the objective(s) or question(s) the review addresses.	Introduction, paragraph four
**METHODS**			
Eligibility criteria	5	Specify the inclusion and exclusion criteria for the review and how studies were grouped for the syntheses.	Materials and methods, 2.3
Information sources	6	Specify all databases, registers, websites, organisations, reference lists and other sources searched or consulted to identify studies. Specify the date when each source was last searched or consulted.	Materials and methods, 2.2
Search strategy	7	Present the full search strategies for all databases, registers and websites, including any filters and limits used.	Materials and methods, 2.2 and Supplementary Table S2
Selection process	8	Specify the methods used to decide whether a study met the inclusion criteria of the review, including how many reviewers screened each record and each report retrieved, whether they worked independently, and if applicable, details of automation tools used in the process.	Materials and methods, 2.4
Data collection process	9	Specify the methods used to collect data from reports, including how many reviewers collected data from each report, whether they worked independently, any processes for obtaining or confirming data from study investigators, and if applicable, details of automation tools used in the process.	Materials and methods, 2.4
Data items	10a	List and define all outcomes for which data were sought. Specify whether all results that were compatible with each outcome domain in each study were sought (e.g. for all measures, time points, analyses), and if not, the methods	Materials and methods, 2.4

Section and Topic	Item #	Checklist item	Location where item is reported
		used to decide which results to collect.	
	10b	List and define all other variables for which data were sought (e.g. participant and intervention characteristics, funding sources). Describe any assumptions made about any missing or unclear information.	Materials and methods, 2.4
Study risk of bias assessment	11	Specify the methods used to assess risk of bias in the included studies, including details of the tool(s) used, how many reviewers assessed each study and whether they worked independently, and if applicable, details of automation tools used in the process.	Materials and methods, 2.5
Effect measures	12	Specify for each outcome the effect measure(s) (e.g. risk ratio, mean difference) used in the synthesis or presentation of results.	Materials and methods, 2.7
Synthesis methods	13a	Describe the processes used to decide which studies were eligible for each synthesis (e.g. tabulating the study intervention characteristics and comparing against the planned groups for each synthesis (item #5)).	Materials and methods, 2.6
	13b	Describe any methods required to prepare the data for presentation or synthesis, such as handling of missing summary statistics, or data conversions.	Materials and methods, 2.7
	13c	Describe any methods used to tabulate or visually display results of individual studies and syntheses.	Materials and methods, 2.7
	13d	Describe any methods used to synthesize results and provide a rationale for the choice(s). If meta-analysis was performed, describe the model(s), method(s) to identify the presence and extent of statistical heterogeneity, and software package(s) used.	Materials and methods, 2.7
	13e	Describe any methods used to explore possible causes of heterogeneity among study results (e.g. subgroup analysis, meta-regression).	Materials and methods, 2.7
	13f	Describe any sensitivity analyses conducted to assess robustness of the synthesized results.	Materials and methods, 2.7
Reporting bias assessment	14	Describe any methods used to assess risk of bias due to missing results in a synthesis (arising from reporting biases).	Materials and methods, 2.5
Certainty assessment	15	Describe any methods used to assess certainty (or confidence) in the body of evidence for an outcome.	Materials and methods, 2.6
**RESULTS**			

Section and Topic	Item #	Checklist item	Location where item is reported
Study selection	16a	Describe the results of the search and selection process, from the number of records identified in the search to the number of studies included in the review, ideally using a flow diagram.	Results, 3.1 and figure 1
	16b	Cite studies that might appear to meet the inclusion criteria, but which were excluded, and explain why they were excluded.	Results, 3.1
Study characteristics	17	Cite each included study and present its characteristics.	Results, 3.2 and Tables1, 2
Risk of bias in studies	18	Present assessments of risk of bias for each included study.	Results, 3.3
Results of individual studies	19	For all outcomes, present, for each study: (a) summary statistics for each group (where appropriate) and (b) an effect estimate and its precision (e.g. confidence/credible interval), ideally using structured tables or plots.	Results, 3.4 and Figures 3-5
Results of syntheses	20a	For each synthesis, briefly summarise the characteristics and risk of bias among contributing studies.	Results, 3.3
	20b	Present results of all statistical syntheses conducted. If meta-analysis was done, present for each the summary estimate and its precision (e.g. confidence/credible interval) and measures of statistical heterogeneity. If comparing groups, describe the direction of the effect.	Results, 3.4
	20c	Present results of all investigations of possible causes of heterogeneity among study results.	Results, 3.4
	20d	Present results of all sensitivity analyses conducted to assess the robustness of the synthesized results.	Results, 3.5
Reporting biases	21	Present assessments of risk of bias due to missing results (arising from reporting biases) for each synthesis assessed.	Results, 3.6
Certainty of evidence	22	Present assessments of certainty (or confidence) in the body of evidence for each outcome assessed.	Results, 3.7
**DISCUSSION**			
Discussion	23a	Provide a general interpretation of the results in the context of other evidence.	Discussion, paragraph one
	23b	Discuss any limitations of the evidence included in the review.	Discussion, paragraph eight
	23c	Discuss any limitations of the review processes used.	Discussion, paragraph

Section and Topic	Item #	Checklist item	Location where item is reported
			eight
	23d	Discuss implications of the results for practice, policy, and future research.	Conclusion
**OTHER INFORMATION**			
Registration and protocol	24a	Provide registration information for the review, including register name and registration number, or state that the review was not registered.	Materials and methods, 2.1
	24b	Indicate where the review protocol can be accessed, or state that a protocol was not prepared.	Materials and methods, 2.1
	24c	Describe and explain any amendments to information provided at registration or in the protocol.	NA
Support	25	Describe sources of financial or non-financial support for the review, and the role of the funders or sponsors in the review.	Funding
Competing interests	26	Declare any competing interests of review authors.	Conflict of interest
Availability of data, code and other materials	27	Report which of the following are publicly available and where they can be found: template data collection forms; data extracted from included studies; data used for all analyses; analytic code; any other materials used in the review.	Data availability statement

From: Page MJ, McKenzie JE, Bossuyt PM, Boutron I, Hoffmann TC, Mulrow CD, et al. The PRISMA 2020 statement: an updated guideline for reporting systematic reviews. BMJ 2021;372:n71. doi: 10.1136/bmj.n71. This work is licensed under CC BY 4.0. To view a copy of this license, visit https://creativecommons.org/licenses/by/4.0/

Table S2: Search strategy

Database	Controlled Vocabulary	Boolean Query Example
PubMed	MeSH:Multiple Sclerosis;transcranial magnetic stimulation;transcranial electrical stimulation	("multiple sclerosis"[MeSH Terms] OR "multiple sclerosis"[Title/Abstract] OR "MS"[Title/Abstract]) AND ("non invasive brain stimulation"[Title/Abstract] OR "NIBS"[Title/Abstract] OR "transcranial magnetic stimulation"[MeSH Terms] OR "transcranial magnetic stimulation"[Title/Abstract] OR "TMS"[Title/Abstract] OR "single pulse transcranial magnetic stimulation"[Title/Abstract] OR "sTMS"[Title/Abstract] OR "repetitive transcranial magnetic stimulation"[Title/Abstract] OR "rTMS"[Title/Abstract] OR "theta burst stimulation"[Title/Abstract] OR "TBS"[Title/Abstract] OR "transcranial direct current stimulation"[MeSH Terms] OR "transcranial electrical stimulation"[Title/Abstract] OR "TES"[Title/Abstract] OR "transcranial direct current stimulation"[Title/Abstract] OR "tDCS"[Title/Abstract] OR "transcranial alternating current stimulation"[Title/Abstract] OR "tACS"[Title/Abstract] OR "transcranial random noise stimulation"[Title/Abstract] OR "tRNS"[Title/Abstract] OR "transcranial focused ultrasound stimulation"[Title/Abstract] OR "tFUS"[Title/Abstract]) AND ("balance"[Title/Abstract] OR "gait"[Title/Abstract] OR "walking"[Title/Abstract] OR "motor function"[Title/Abstract] OR "posture control"[Title/Abstract])
Embase	Rmtree:multiple sclerosis;transcranial magnetic stimulation;Repetitive transcranial magnetic stimulation;transcranial electrical stimulation;transcranial direct current stimulation;transcranial alternating current stimulation;transcranial random noise stimulation;gait	('multiple sclerosis'/exp OR 'multiple sclerosis':ab,ti OR ms:ab,ti) AND ('non-invasive brain stimulation':ab,ti OR nibs:ab,ti OR 'transcranial magnetic stimulation'/exp OR 'transcranial magnetic stimulation':ab,ti OR tms:ab,ti OR 'single pulse transcranial magnetic stimulation':ab,ti OR stms:ab,ti OR 'repetitive transcranial magnetic stimulation'/exp OR 'repetitive transcranial magnetic stimulation':ab,ti OR rtms:ab,ti OR 'theta-burst stimulation':ab,ti OR tbs:ab,ti OR 'transcranial electrical stimulation'/exp OR 'transcranial electrical stimulation':ab,ti OR tes:ab,ti OR 'transcranial direct current stimulation'/exp OR 'transcranial direct current stimulation':ab,ti OR tdcs:ab,ti OR 'transcranial alternating current stimulation'/exp OR 'transcranial alternating current stimulation':ab,ti OR tacs:ab,ti OR 'transcranial random noise stimulation'/exp OR 'transcranial random noise stimulation':ab,ti OR trns:ab,ti OR 'transcranial focused ultrasound stimulation':ab,ti OR tfus:ab,ti) AND (balance:ab,ti OR 'gait'/exp OR gait:ab,ti OR walking:ab,ti OR 'motor function':ab,ti OR 'posture

	control':ab,ti)
Web of Science	(TS=(multiple sclerosis) OR TS=(MS)) AND (TS=(non-invasive brain stimulation) OR TS=(NIBS) OR TS=(transcranial magnetic stimulation) OR TS=(TMS) OR TS=(single pulse transcranial magnetic stimulation) OR TS=(sTMS) OR TS=(Repetitive transcranial magnetic stimulation) OR TS=(rTMS) OR TS=(Theta-burst stimulation) OR TS=(TBS) OR TS=(transcranial electrical stimulation) OR TS=(TES) OR TS=(transcranial direct current stimulation) OR TS=(tDCS) OR TS=(transcranial alternating current stimulation) OR TS=(tACS) OR TS=(transcranial random noise stimulation) OR TS=(tRNS) OR TS=(transcranial focused ultrasound stimulation) OR TS=(tFUS)) AND (TS=(balance) OR TS=(gait) OR TS=(walking) OR TS=(motor function) OR TS=(posture control))
Scopus	(TITLE-ABS-KEY ("multiple sclerosis*") AND TITLE-ABS-KEY ("non-invasive brain stimulation*" OR "transcranial magnetic stimulation*" OR "single pulse transcranial magnetic stimulation*" OR "Repetitive transcranial magnetic stimulation*" OR "Theta-burst stimulation*" OR "transcranial electrical stimulation*" OR "transcranial direct current stimulation*" OR "transcranial alternating current stimulation*" OR "transcranial random noise stimulation*" OR "transcranial focused ultrasound stimulation*") AND TITLE-ABS-KEY ("balance" OR "gait" OR "walking" OR "motor function*" OR "posture control*"))
Cochrane	(multiple sclerosis OR MS):ti,ab,kw AND (non-invasive brain stimulation OR NIBS OR transcranial magnetic stimulation OR TMS OR single pulse transcranial magnetic stimulation OR sTMS OR Repetitive transcranial magnetic stimulation OR rTMS OR Theta-burst stimulation OR TBS OR transcranial electrical stimulation OR TES OR transcranial direct current stimulation OR tDCS OR transcranial alternating current stimulation OR tACS OR transcranial random noise stimulation OR tRNS OR transcranial focused ultrasound stimulation OR tFUS):ti,ab,kw AND (balance OR gait OR walking OR motor function OR posture control):ti,ab,kw
CNKI Scholar	SU = (Multiple Sclerosis + MS) AND SU = (Non-invasive Brain Stimulation + NIBS + Transcranial Magnetic Stimulation + TMS + Single-pulse TMS + Repetitive TMS + Theta Burst Stimulation + Transcranial Electrical Stimulation

	+ TES + Transcranial Direct Current Stimulation + Transcranial Alternating Current Stimulation + Transcranial Random Noise Stimulation + Transcranial Focused Ultrasound Stimulation)
ClinicalTrials.gov	Condition or disease: "Multiple Sclerosis" OR "MS" Other terms: ("non-invasive brain stimulation" OR NIBS OR "transcranial magnetic stimulation" OR TMS OR "re petitive transcranial magnetic stimulation" OR rTMS OR "theta burst stimulation" OR TBS OR "transcranial dire ct current stimulation" OR tDCS OR "transcranial alterna ting current stimulation" OR tACS OR "transcranial rand om noise stimulation" OR tRNS OR "transcranial focuse d ultrasound stimulation" OR tFUS) Filters: Study type: Interventional Studies (Clinical Trials)

Figure S1:Forest plot of subgroup analysis based on NIBS type of TUG.

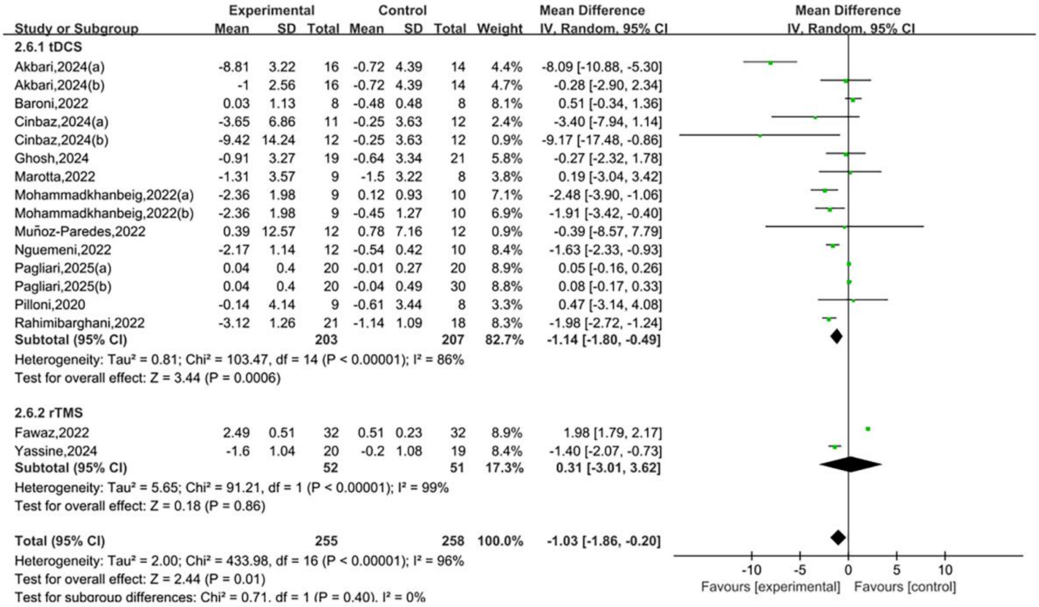

Figure S2:Forest plot of subgroup analysis based on MS subtype of TUG.

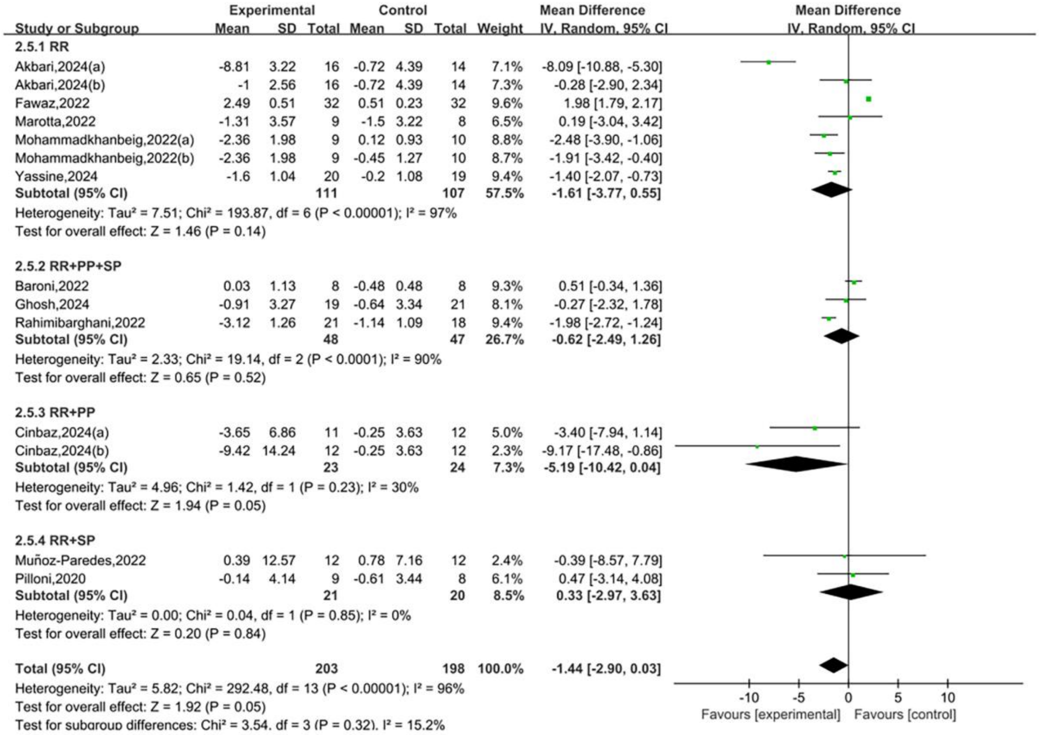

Figure S3:Forest plot for subgroup analysis based on intervention duration of TUG.

	Experimental			Control				Mean Difference
Study or Subgroup	Mean	SD	Total	Mean	SD	Total	Weight	IV, Random, 95% CI
**2.2.1 2 weeks**								
Baroni,2022	0.03	1.13	8	-0.48	0.48	8	8.4%	0.51 [-0.34, 1.36]
Fawaz,2022	2.49	0.51	32	0.51	0.23	32	9.2%	1.98 [1.79, 2.17]
Marotta,2022	-1.31	3.57	9	-1.5	3.22	8	3.9%	0.19 [-3.04, 3.42]
Nguemeni,2022	-2.17	1.14	12	-0.54	0.42	10	8.7%	-1.63 [-2.33, -0.93]
**Subtotal (95% CI)**			**61**			**58**	**30.2%**	**0.28 [-1.83, 2.39]**
Heterogeneity: Tau² = 4.07; Chi² = 104.23, df = 3 (P < 0.00001); I² = 97%								
Test for overall effect: Z = 0.26 (P = 0.79)								
**2.2.2 4 weeks**								
Akbari,2024(a)	-8.81	3.22	16	-0.72	4.39	14	4.6%	-8.09 [-10.88, -5.30]
Akbari,2024(b)	-1	2.56	16	-0.72	4.39	14	4.9%	-0.28 [-2.90, 2.34]
Cinbaz,2024(a)	-3.65	6.86	11	-0.25	3.63	12	2.5%	-3.40 [-7.94, 1.14]
Cinbaz,2024(b)	-9.42	14.24	12	-0.25	3.63	12	0.9%	-9.17 [-17.48, -0.86]
Muñoz-Paredes,2022	0.39	12.57	12	0.78	7.16	12	1.0%	-0.39 [-8.57, 7.79]
Yassine,2024	-1.6	1.04	20	-0.2	1.08	19	8.7%	-1.40 [-2.07, -0.73]
**Subtotal (95% CI)**			**87**			**83**	**22.5%**	**-3.42 [-6.32, -0.53]**
Heterogeneity: Tau² = 8.44; Chi² = 25.80, df = 5 (P < 0.0001); I² = 81%								
Test for overall effect: Z = 2.32 (P = 0.02)								
**2.2.3 6 weeks**								
Ghosh,2024	-0.91	3.27	19	-0.64	3.34	21	6.0%	-0.27 [-2.32, 1.78]
Mohammadkhanbeig,2022(a)	-2.36	1.98	9	0.12	0.93	10	7.3%	-2.48 [-3.90, -1.06]
Mohammadkhanbeig,2022(b)	-2.36	1.98	9	-0.45	1.27	10	7.1%	-1.91 [-3.42, -0.40]
Pagliari,2025(a)	0.04	0.4	20	-0.01	0.27	20	9.2%	0.05 [-0.16, 0.26]
Pagliari,2025(b)	0.04	0.4	20	-0.04	0.49	30	9.1%	0.08 [-0.17, 0.33]
Rahimibarghani,2022	-3.12	1.26	21	-1.14	1.09	18	8.6%	-1.98 [-2.72, -1.24]
**Subtotal (95% CI)**			**98**			**109**	**47.3%**	**-0.88 [-1.55, -0.21]**
Heterogeneity: Tau² = 0.45; Chi² = 45.22, df = 5 (P < 0.00001); I² = 89%								
Test for overall effect: Z = 2.59 (P = 0.010)								
**Total (95% CI)**			**246**			**250**	**100.0%**	**-1.08 [-1.92, -0.24]**
Heterogeneity: Tau² = 2.00; Chi² = 433.98, df = 15 (P < 0.00001); I² = 97%								
Test for overall effect: Z = 2.51 (P = 0.01)								
Test for subgroup differences: Chi² = 4.14, df = 2 (P = 0.13), I² = 51.7%								

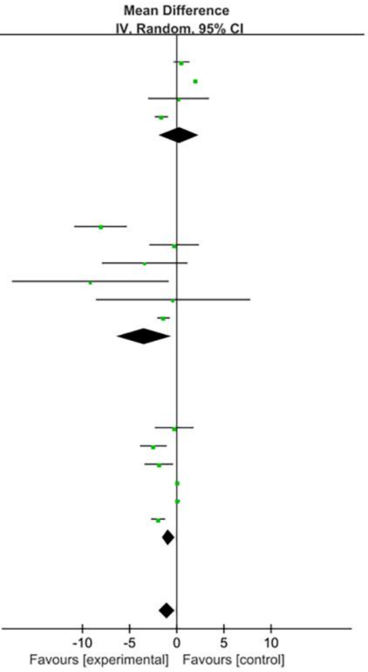

Figure S4:Forest plot for subgroup analysis based on stimulation intensity of TUG.

	Experimental			Control				Mean Difference
Study or Subgroup	Mean	SD	Total	Mean	SD	Total	Weight	IV, Random, 95% CI
**2.1.1 1.5mA**								
Akbari,2024(a)	-8.81	3.22	16	-0.72	4.39	14	5.4%	-8.09 [-10.88, -5.30]
Akbari,2024(b)	-1	2.56	16	-0.72	4.39	14	5.7%	-0.28 [-2.90, 2.34]
Rahimibarghani,2022	-3.12	1.26	21	-1.14	1.09	18	10.3%	-1.98 [-2.72, -1.24]
**Subtotal (95% CI)**			**53**			**46**	**21.4%**	**-3.34 [-7.00, 0.32]**
Heterogeneity: Tau² = 9.22; Chi² = 19.53, df = 2 (P < 0.0001); I² = 90%								
Test for overall effect: Z = 1.79 (P = 0.07)								
**2.1.2 2mA**								
Baroni,2022	0.03	1.13	8	-0.48	0.48	8	10.1%	0.51 [-0.34, 1.36]
Cinbaz,2024(a)	-3.65	6.86	11	-0.25	3.63	12	2.9%	-3.40 [-7.94, 1.14]
Cinbaz,2024(b)	-9.42	14.24	12	-0.25	3.63	12	1.1%	-9.17 [-17.48, -0.86]
Fawaz,2022	2.49	0.51	32	0.51	0.23	32	11.0%	1.98 [1.79, 2.17]
Ghosh,2024	-0.91	3.27	19	-0.64	3.34	21	7.0%	-0.27 [-2.32, 1.78]
Marotta,2022	-1.31	3.57	9	-1.5	3.22	8	4.6%	0.19 [-3.04, 3.42]
Mohammadkhanbeig,2022(a)	-2.36	1.98	9	0.12	0.93	10	8.7%	-2.48 [-3.90, -1.06]
Muñoz-Paredes,2022	0.39	12.57	12	0.78	7.16	12	1.1%	-0.39 [-8.57, 7.79]
Nguemeni,2022	-2.17	1.14	12	-0.54	0.42	10	10.4%	-1.63 [-2.33, -0.93]
Pagliari,2025(a)	0.04	0.4	20	-0.01	0.27	20	11.0%	0.05 [-0.16, 0.26]
Pagliari,2025(b)	0.04	0.4	20	-0.04	0.49	30	10.9%	0.08 [-0.17, 0.33]
**Subtotal (95% CI)**			**164**			**175**	**78.6%**	**-0.35 [-1.29, 0.58]**
Heterogeneity: Tau² = 1.56; Chi² = 299.78, df = 10 (P < 0.00001); I² = 97%								
Test for overall effect: Z = 0.74 (P = 0.46)								
**Total (95% CI)**			**217**			**221**	**100.0%**	**-0.97 [-1.87, -0.06]**
Heterogeneity: Tau² = 1.92; Chi² = 388.58, df = 13 (P < 0.00001); I² = 97%								
Test for overall effect: Z = 2.10 (P = 0.04)								
Test for subgroup differences: Chi² = 2.40, df = 1 (P = 0.12), I² = 58.3%								

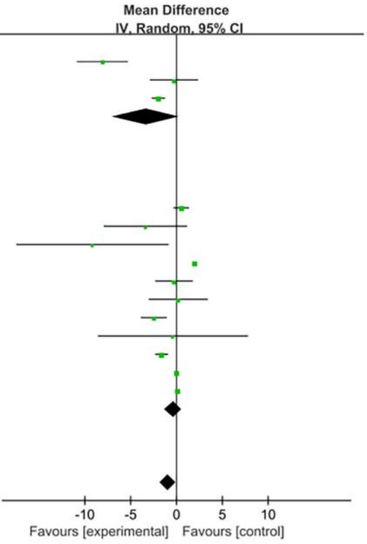

Figure S5:Forest plot for subgroup analysis based on stimulation site of TUG.

Study or Subgroup	Experimental Mean	Experimental SD	Experimental Total	Control Mean	Control SD	Control Total	Weight	Mean Difference IV, Random, 95% CI
**2.3.1 cerebellum**								
Akbari,2024(a)	-8.81	3.22	16	-0.72	4.39	14	4.4%	-8.09 [-10.88, -5.30]
Nguemeni,2022	-2.17	1.14	12	-0.54	0.42	10	13.3%	-1.63 [-2.33, -0.93]
**Subtotal (95% CI)**			**28**			**24**	**17.7%**	**-4.71 [-11.04, 1.61]**
Heterogeneity: Tau² = 19.79; Chi² = 19.41, df = 1 (P < 0.0001); I² = 95%								
Test for overall effect: Z = 1.46 (P = 0.14)								
**2.3.2 left DLPFC**								
Akbari,2024(b)	-1	2.56	16	-0.72	4.39	14	4.8%	-0.28 [-2.90, 2.34]
Muñoz-Paredes,2022	0.39	12.57	12	0.78	7.16	12	0.7%	-0.39 [-8.57, 7.79]
Pagliari,2025(a)	0.04	0.4	20	-0.01	0.27	20	15.1%	0.05 [-0.16, 0.26]
Pagliari,2025(b)	0.04	0.4	20	-0.04	0.49	30	15.0%	0.08 [-0.17, 0.33]
**Subtotal (95% CI)**			**68**			**76**	**35.7%**	**0.06 [-0.10, 0.22]**
Heterogeneity: Tau² = 0.00; Chi² = 0.11, df = 3 (P = 0.99); I² = 0%								
Test for overall effect: Z = 0.75 (P = 0.46)								
**2.3.3 M1（C3)**								
Cinbaz,2024(a)	-3.65	6.86	11	-0.25	3.63	12	2.0%	-3.40 [-7.94, 1.14]
Marotta,2022	-1.31	3.57	9	-1.5	3.22	8	3.6%	0.19 [-3.04, 3.42]
Pilloni,2020	-0.14	4.14	9	-0.61	3.44	8	3.0%	0.47 [-3.14, 4.08]
Rahimibarghani,2022	-3.12	1.26	21	-1.14	1.09	18	13.1%	-1.98 [-2.72, -1.24]
**Subtotal (95% CI)**			**50**			**46**	**21.7%**	**-1.54 [-2.79, -0.30]**
Heterogeneity: Tau² = 0.43; Chi² = 3.68, df = 3 (P = 0.30); I² = 19%								
Test for overall effect: Z = 2.43 (P = 0.01)								
**2.3.4 M1（Cz)**								
Ghosh,2024	-0.91	3.27	19	-0.64	3.34	21	6.6%	-0.27 [-2.32, 1.78]
Mohammadkhanbeig,2022(a)	-2.36	1.98	9	0.12	0.93	10	9.4%	-2.48 [-3.90, -1.06]
Mohammadkhanbeig,2022(b)	-2.36	1.98	9	-0.45	1.27	10	8.9%	-1.91 [-3.42, -0.40]
**Subtotal (95% CI)**			**37**			**41**	**24.9%**	**-1.74 [-2.90, -0.58]**
Heterogeneity: Tau² = 0.36; Chi² = 3.04, df = 2 (P = 0.22); I² = 34%								
Test for overall effect: Z = 2.95 (P = 0.003)								
**Total (95% CI)**			**183**			**187**	**100.0%**	**-1.30 [-2.00, -0.60]**
Heterogeneity: Tau² = 0.82; Chi² = 96.51, df = 12 (P < 0.00001); I² = 88%								
Test for overall effect: Z = 3.66 (P = 0.0003)								
Test for subgroup differences: Chi² = 17.29, df = 3 (P = 0.0006), I² = 82.7%								

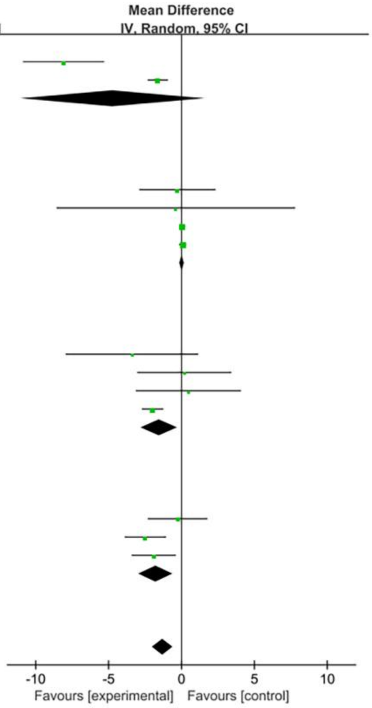

Figure S6:Forest plot of subgroup analysis based on NIBS type of BBS.

Study or Subgroup	Experimental Mean	Experimental SD	Experimental Total	Control Mean	Control SD	Control Total	Weight	Mean Difference IV, Random, 95% CI
**3.6.1 tDCS**								
Akbari,2024(a)	17.43	7.22	16	1.36	8.3	14	7.0%	16.07 [10.46, 21.68]
Akbari,2024(b)	2.44	8.03	16	1.36	8.3	14	6.7%	1.08 [-4.78, 6.94]
Charehjou,2024	3.2	2.4	10	4.2	2.46	10	12.1%	-1.00 [-3.13, 1.13]
Ehsani,2022	8.55	1.65	10	4.77	1.46	10	13.1%	3.78 [2.41, 5.15]
Ghosh,2024	2.09	5.05	19	1.22	4.8	21	10.7%	0.87 [-2.19, 3.93]
Marotta,2022	4.97	6.41	9	1	3.89	8	7.8%	3.97 [-1.01, 8.95]
Mohammadkhanbeig,2022(a)	5.33	4.73	9	0	5.97	10	8.1%	5.33 [0.51, 10.15]
Mohammadkhanbeig,2022(b)	5.33	4.73	9	3.3	4.55	10	9.0%	2.03 [-2.15, 6.21]
**Subtotal (95% CI)**			**98**			**97**	**74.6%**	**3.57 [0.84, 6.31]**
Heterogeneity: Tau² = 11.40; Chi² = 38.99, df = 7 (P < 0.00001); I² = 82%								
Test for overall effect: Z = 2.56 (P = 0.01)								
**3.6.2 rTMS**								
Mohamed,2025	4.5	3.72	15	2.6	3	15	11.7%	1.90 [-0.52, 4.32]
Yassine,2024	7.2	0.79	20	1.3	0.83	19	13.7%	5.90 [5.39, 6.41]
**Subtotal (95% CI)**			**35**			**34**	**25.4%**	**4.08 [0.18, 7.99]**
Heterogeneity: Tau² = 7.20; Chi² = 10.06, df = 1 (P = 0.002); I² = 90%								
Test for overall effect: Z = 2.05 (P = 0.04)								
**Total (95% CI)**			**133**			**131**	**100.0%**	**3.62 [1.50, 5.75]**
Heterogeneity: Tau² = 8.50; Chi² = 77.43, df = 9 (P < 0.00001); I² = 88%								
Test for overall effect: Z = 3.35 (P = 0.0008)								
Test for subgroup differences: Chi² = 0.04, df = 1 (P = 0.83), I² = 0%								

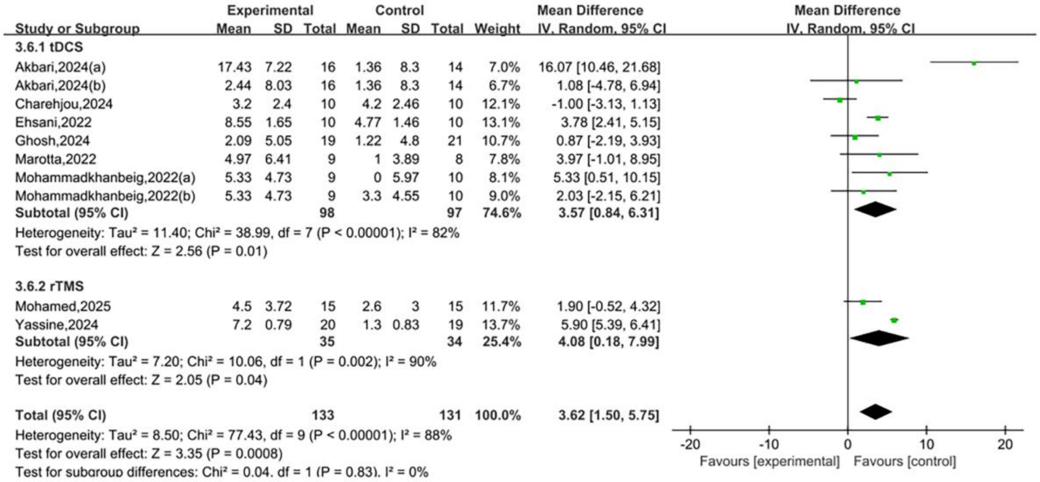

Figure S7:Forest plot of subgroup analysis based on MS subtype of BBS.

Study or Subgroup	Experimental Mean	Experimental SD	Experimental Total	Control Mean	Control SD	Control Total	Weight	Mean Difference IV, Random, 95% CI
**3.5.1 RR**								
Akbari,2024(a)	17.43	7.22	16	1.36	8.3	14	9.7%	16.07 [10.46, 21.68]
Akbari,2024(b)	2.44	8.03	16	1.36	8.3	14	9.3%	1.08 [-4.78, 6.94]
Marotta,2022	4.97	6.41	9	1	3.89	8	10.8%	3.97 [-1.01, 8.95]
Mohammadkhanbeig,2022(a)	5.33	4.73	9	0	5.97	10	11.0%	5.33 [0.51, 10.15]
Mohammadkhanbeig,2022(b)	5.33	4.73	9	3.3	4.55	10	12.2%	2.03 [-2.15, 6.21]
Yassine,2024	7.2	0.79	20	1.3	0.83	19	18.1%	5.90 [5.39, 6.41]
**Subtotal (95% CI)**			**79**			**75**	**71.2%**	**5.61 [2.57, 8.65]**
Heterogeneity: Tau² = 9.42; Chi² = 19.14, df = 5 (P = 0.002); I² = 74%								
Test for overall effect: Z = 3.62 (P = 0.0003)								
**3.5.2 RR+SP**								
Mohamed,2025	4.5	3.72	15	2.6	3	15	15.7%	1.90 [-0.52, 4.32]
Tramontano,2020	4.67	3.06	8	4.23	4.45	8	13.1%	0.44 [-3.30, 4.18]
**Subtotal (95% CI)**			**23**			**23**	**28.8%**	**1.47 [-0.56, 3.50]**
Heterogeneity: Tau² = 0.00; Chi² = 0.41, df = 1 (P = 0.52); I² = 0%								
Test for overall effect: Z = 1.42 (P = 0.16)								
**Total (95% CI)**			**102**			**98**	**100.0%**	**4.35 [1.80, 6.90]**
Heterogeneity: Tau² = 9.24; Chi² = 36.53, df = 7 (P < 0.00001); I² = 81%								
Test for overall effect: Z = 3.35 (P = 0.0008)								
Test for subgroup differences: Chi² = 4.93, df = 1 (P = 0.03), I² = 79.7%								

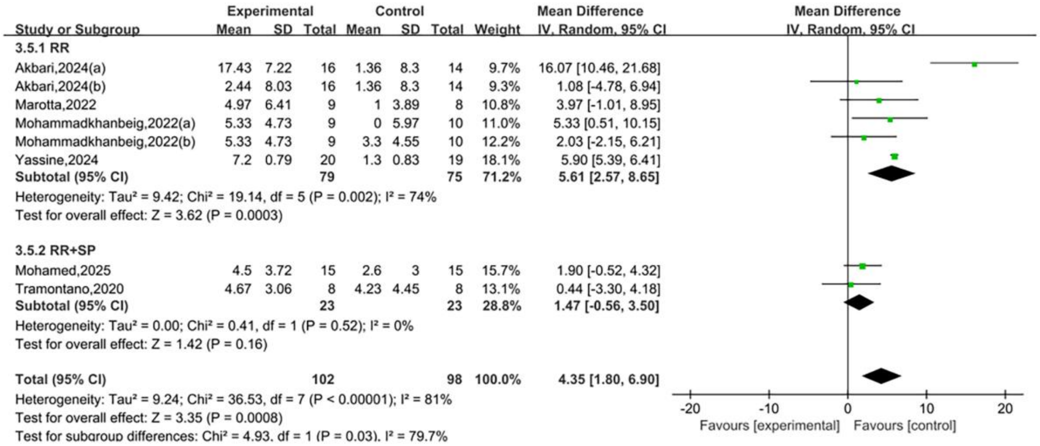

Figure S8:Forest plot for subgroup analysis based on intervention duration of BBS.

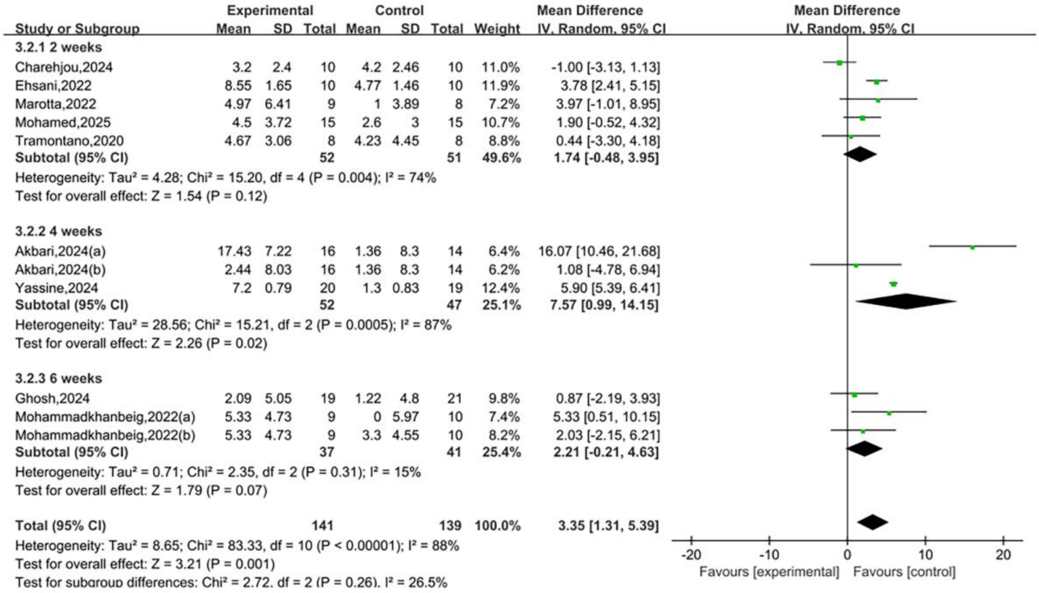

Figure S9:Forest plot for subgroup analysis based on stimulation intensity of BBS.

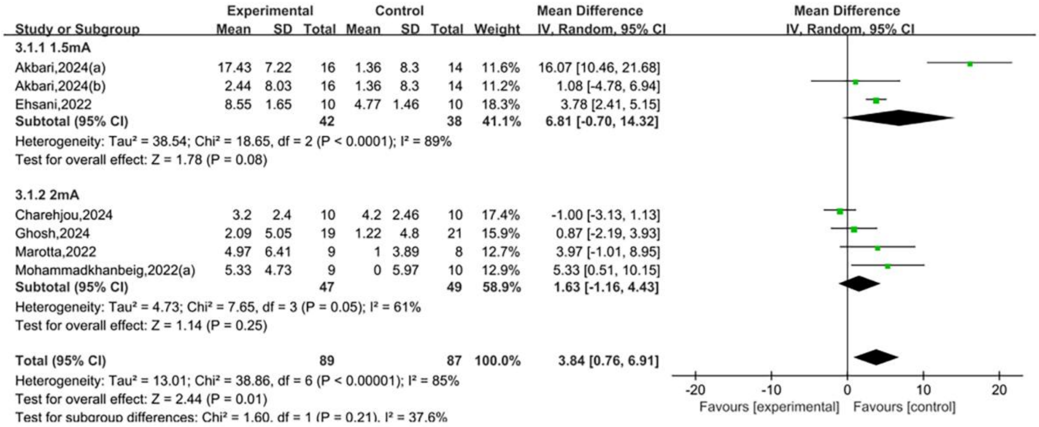

Figure S10:Forest plot for subgroup analysis based on stimulation site of BBS.

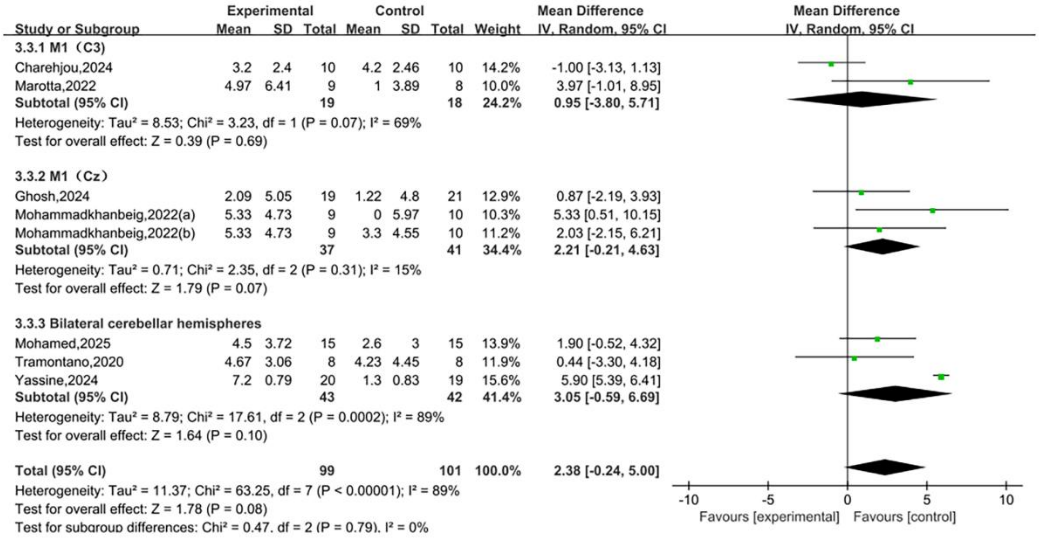

Supplement: Supplementary file 1 [file Data_Sheet_1.pdf]
